# Principles underlying the input-dependent formation and organization of memories

**DOI:** 10.1162/netn_a_00086

**Published:** 2019-05-01

**Authors:** Juliane Herpich, Christian Tetzlaff

**Affiliations:** Department of Computational Neuroscience, Third Institute of Physics - Biophysics, Georg-August-University, Göttingen, Germany; Bernstein Center for Computational Neuroscience, Georg-August-University, Göttingen, Germany; Department of Computational Neuroscience, Third Institute of Physics - Biophysics, Georg-August-University, Göttingen, Germany; Bernstein Center for Computational Neuroscience, Georg-August-University, Göttingen, Germany

**Keywords:** Synaptic plasticity, Memory, Memory interaction, Synaptic scaling, Inhibition

## Abstract

The neuronal system exhibits the remarkable ability to dynamically store and organize incoming information into a web of memory representations (items), which is essential for the generation of complex behaviors. Central to memory function is that such memory items must be (1) discriminated from each other, (2) associated to each other, or (3) brought into a sequential order. However, how these three basic mechanisms are robustly implemented in an input-dependent manner by the underlying complex neuronal and synaptic dynamics is still unknown. Here, we develop a mathematical framework, which provides a direct link between different synaptic mechanisms, determining the neuronal and synaptic dynamics of the network, to create a network that emulates the above mechanisms. Combining correlation-based synaptic plasticity and homeostatic synaptic scaling, we demonstrate that these mechanisms enable the reliable formation of sequences and associations between two memory items still missing the capability for discrimination. We show that this shortcoming can be removed by additionally considering inhibitory synaptic plasticity. Thus, the here-presented framework provides a new, functionally motivated link between different known synaptic mechanisms leading to the self-organization of fundamental memory mechanisms.

## Author Summary

Higher-order animals are permanently exposed to a variety of environmental inputs that have to be processed and stored such that the animal can react appropriately. Thereby, the ongoing challenge for the neuronal system is to continuously store novel and meaningful stimuli and, dependent on their content, to integrate them into the existing web of knowledge or memories. The smallest organizational entity of such a web of memories is described by the functional relation of two interconnected memories: they can be either unrelated (discrimination), mutually related (association), or unidirectionally related (sequence). However, the neuronal and synaptic dynamics underlying the formation of such structures are mainly unknown. To investigate possible links between physiological mechanisms and the organization of memories, in this work, we develop a general mathematical framework enabling an analytical approach. Thereby, we show that the well-known mechanisms of synaptic plasticity and homeostatic scaling in conjunction with inhibitory synaptic plasticity enables the reliable formation of all basic relations between two memories. This work provides a further step in the understanding of the complex dynamics underlying the organization of knowledge in neural systems.

## INTRODUCTION

Learning and memorizing various pieces of information from the environment are vital functions for the survival of living beings. In addition, the corresponding neuronal system has to learn the environmental relations between these different pieces. For this, the neuronal system has to form memory representations of the information and to organize them accordingly. However, the neuronal and synaptic dynamics determining the organization of these representations are widely unknown.

The synaptic-plasticity-and-memory hypothesis relates the formation of memory representations to the underlying neuronal and synaptic mechanisms (Martin, Grimwood, & Morris, 2000; Martin & Morris, 2002). Namely, a to-be-learned piece of information activates via an environmental stimulus a certain population of neurons triggering synaptic plasticity.Synaptic plasticity, in turn, changes the weights of the synapses between the activated neurons such that these neurons become strongly interconnected and form a memory representation—so-called Hebbian cell assembly (CA)—of the presented information (Hebb, 1949; Palm, 1981; Buzsaki, 2010; Palm, Knoblauch, Hauser, & Schütz, 2014). Besides the formation of a memory representation, the newly learned piece of information is also related to already stored information (Hebb, 1949; Wickelgren, 1999; Tse et al., 2007, 2011). Thereby, the relations or functional organizations between different memory representations can be organized in three different, fundamental ways: they can be unrelated (discrimination), mutually related (association), or unidirectionally related (sequence). However, although the link between the formation of a single memory representation and the underlying neuronal and synaptic mechanisms is already well established (Garagnani, Wennekers, & Pulvermüller, 2009; Tetzlaff, Kolodziejski, Timme, Tsodyks, & Wörgötter, 2013; Litwin-Kumar & Doiron, 2014; Zenke, Agnes, & Gerstner, 2015), it is largely unknown which mechanisms enable the self-organized formation of relations *between* memory representations.

In this theoretical study, we have developed the first theoretical framework enabling one to analyze the ability of diverse neuronal and synaptic mechanisms to form memory representations and, in addition, to form the different types of memory-relations. Thereby, our analysis indicates that the interaction of correlation-based synaptic plasticity with homeostatic synaptic scaling is not sufficient to form all types of memory relations, although it enables the formation of individual memory representations (Tetzlaff et al., 2013; Tetzlaff, Dasgupta, Kulvicius, & Wörgötter, 2015). However, our analysis shows that, if the average level of inhibition within the memory representations is significantly lower than the average level in the remaining network, the neuronal system is able, on the one hand, to form memory representations and, on the other hand, to organize them into the fundamental types of memory relations in an input-dependent, self-organized manner.

Several theoretical studies (Tetzlaff et al., 2013; Litwin-Kumar & Doiron, 2014; Zenke et al., 2015; Tetzlaff et al., 2015; Chenkov, Sprekeler, & Kempter, 2017) investigated the formation of individual memory representations in neuronal systems indicating correlation-based synaptic plasticity as essential mechanism. In addition, homeostatic plasticity, as synaptic scaling (Turrigiano, Leslie, Desai, Rutherford, & Nelson, 1998), is required to keep the system in an adequate dynamic regime (Dayan & Abbott, 2001; Tetzlaff, Kolodziejski, Timme, & Wörgötter, 2011; Zenke, Hennequin, & Gerstner, 2013). Further studies indicate that synaptic plasticity and homeostatic plasticity also yield the formation of sequences of representations (Chenkov et al., 2017; Lazar, Pipa, & Triesch, 2009; Tully, Lindn, Hennig, & Lansner, 2016). However, it remains unclear whether the interaction of synaptic and homeostatic plasticity also enables the formation of further memory relations as described above. Interestingly, several theoretical studies (Wickelgren, 1999; Palm, 1982; Byrne & Huyck, 2010) indicate that a neural system with the ability to form all described memory relations has an algorithmic advantage to process the stored information. Furthermore, the neuronal dynamics resulting from interconnected memory representations match experimental results on the psychological (Romani, Pinkoviezky, Rubin, & Tsodyks, 2013) and single-neuron level (Griniasty, Tsodyks, & Amit, 1993; Amit, Brunel, & Tsodyks, 1994). However, these studies consider neural systems after completed learning; thus, it is unclear how neuronal systems form the required relations between memory representations in a self-organized manner.

We consider a neuronal network model with plastic excitatory connections, which are governed by the interaction of correlation-based and homeostatic plasticity. As already shown in previous studies, this interaction enables the self-organized formation of individual memory representations (Tetzlaff et al., 2013, 2015). Similar to these studies, we use methods from the scientific field of nonlinear dynamics (Glendinning, 1994; Izhikevich, 2007) to derive the underlying mechanisms yielding the self-organized formation of the relations between memory representations. Thus, we analyze the ability of the plastic network to form different types of relations between two memory representations—namely, discrimination, sequences, and association. Please note that this is a high-dimensional problem of the order of *N*^2^ (given *N* neurons). To reduce complexity, standard approaches such as mean-field analysis are not feasible, as they obliterate the different memory representations involved. Thus, we developed a new theoretical framework by considering the mean equilibrium states of the relevant system variables and by comparing them to constraints given for the different memory-relations. Thereby, we map the constraints on the long-term average activity level of the neuronal populations involved, reducing the problem to a two-dimensional one, which can be analyzed graphically and analytically. By this framework, we optimized the parameters of the system and identified that correlation-based and homeostatic plasticity do not suffice to form all three types of memory relation. Instead, if the average inhibitory level within the memory representations is below control level, memory representations can be formed, maintained, and related to each other. In addition, we show that the required state can also be reached in a self-organized, dynamic way by the interplay between excitatory and inhibitory synaptic plasticity. Thus, the here-presented results provide a next step to understanding the complex dynamics underlying the formation of memory relations in neuronal networks.

## RESULTS

**Figure 1.**
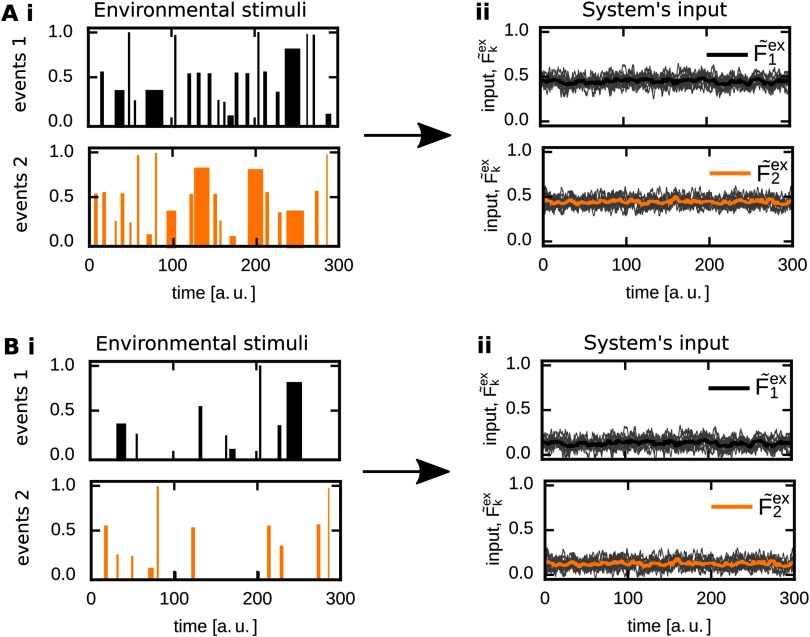
Given that the synaptic changes are slow, the average input determines the synaptic dynamics. (A) Individual presentations or events of one specific input shown to a corresponding population of neurons can have different duration, amplitude, and probability of occurrence (i). However, compared with the duration of a single event, synaptic changes are in general slow. Therefore, the average input strength (ii), considered here, mainly influences the long-term dynamics of the neuronal system (Figure 2A). (B) Thus, different duration, amplitude, and/or probability of occurrence map to a different average input triggering different synaptic dynamics.

In our work, we analyze the ability of two neuronal populations *p* ∈{1, 2} to become memory representations and, in parallel, to reliably build up different functional organizations such as discrimination, sequence, and association (Table 1). In general, the external input to population *p* should trigger synaptic changes within the population such that it becomes a memory representation of its specific input. Individual input events can have different amplitude, duration, and probability of occurrence (Figure 1Ai); however, synaptic changes are slow compared with the presentation of single input events such that the average over all input events determines the formation of a memory representation (Figure 1Aii). Thus, throughout this study, we consider the average input stimulation a population receives, whereby a reduced number of input events and/or reduced amplitudes and shorter durations map to a lower average input (compare Figure 1, A with B).

**Table 1.** Synaptic weight-dependent definition of memory and different forms of functional organization of two interconnected neuronal populations. The different functional organizations are defined based on the average excitatory synaptic weights $\langle {\tilde{\omega }}_{11}\rangle$, $\langle {\tilde{\omega }}_{22}\rangle$, $\langle {\tilde{\omega }}_{21}\rangle$, and $\langle {\tilde{\omega }}_{12}\rangle$ at equilibrium in relation to the average inhibitory synaptic weights ($\tilde{\theta }$).

**Memory representation**	$\langle {\tilde{\omega }}_{11}\rangle$	$\langle {\tilde{\omega }}_{22}\rangle$	$\langle {\tilde{\omega }}_{21}\rangle$	$\langle {\tilde{\omega }}_{12}\rangle$	**functional organization**	**abbreviation**	**color code**
✗	$<\tilde{\theta }$	$<\tilde{\theta }$	−	−	none	nm	grey
							
✓	$>\tilde{\theta }$	$>\tilde{\theta }$	$<\tilde{\theta }$	$<\tilde{\theta }$	discrimination	disc	blue
							
			$<\tilde{\theta }$	$>\tilde{\theta }$	sequence 12	s12	yellow
							
			$>\tilde{\theta }$	$<\tilde{\theta }$	sequence 21	s21	green
							
			$>\tilde{\theta }$	$>\tilde{\theta }$	association	asc	red
							
			bistable		various	bs	pink

Given two populations of neurons, dependent on the input properties, connections between the populations should also be altered to form the neuronal substrate underlying the diverse functional organizations described above. In accordance to the synaptic-plasticity-and-memory hypothesis (Martin et al., 2000; Hebb, 1949), we define a neuronal population as being a *memory representation* if its neurons are strongly interconnected. In other words, the average excitatory synaptic strength between all neurons within the population has to be larger than the average inhibitory synaptic strength. Thus, because of the dominant excitation, neuronal activity within the population will be amplified. We define the relation between two memory representations in a similar manner based on the relation of excitation and inhibition between the corresponding neuronal populations: in general, if the average excitatory synaptic strength from one population to the other is larger than the average inhibitory synaptic strength, an increased level of activity in the former population triggers an increased activation in the latter. This can be different for both directions such that, for instance, the net connection from population 1 to 2 can be excitatory and inhibitory from 2 to 1. This case is defined as a *sequence* from 1 to 2. Similarly, an *association* is present if both connections are excitatory-dominated, and a *discrimination* consists of both directions being zero or inhibition-dominated.

**Figure 2.**
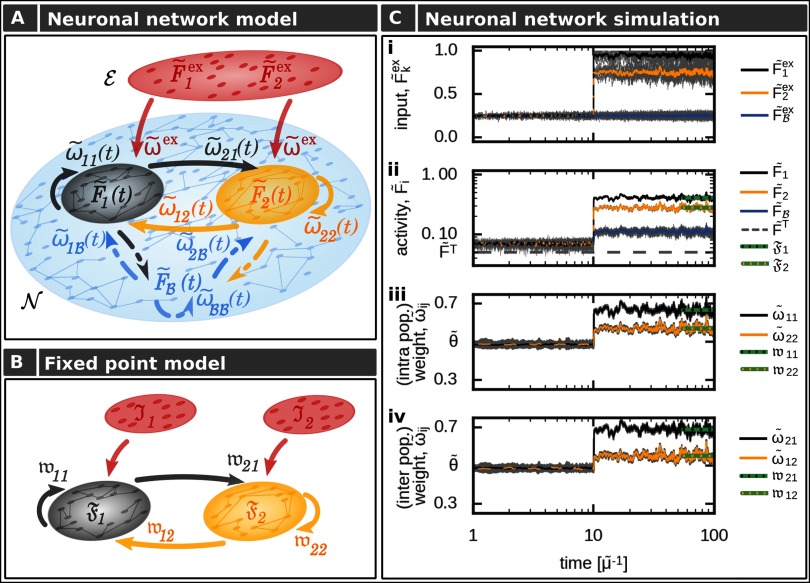
The formation of interconnected memory representations in a plastic neural network. (A) In a recurrent network 𝒩, two neuronal populations (1: black; 2: yellow) receive specific external inputs of average amplitudes ${\tilde{F}}_{1}^{\text{ex}}$ and ${\tilde{F}}_{2}^{\text{ex}}$. All remaining neurons of the network (blue) are combined to a background population ℬ and serve as control neurons receiving noisy external inputs. Each population *p* ∈{1, 2, ℬ} is described by its mean intra-population-synaptic weight ${\tilde{\omega }}_{pp}$, its mean activity ${\tilde{F}}_{p}$, and its connections to other populations (*p′*∈{1, 2, ℬ}∖*p*) via a set of synapses with average synaptic strength ${\tilde{\omega }}_{p^{\prime }p}$. (B) The abstraction of the neuronal network model yields a low-dimensional one described by the mean equilibrium activities (𝔉) and corresponding mean equilibrium synaptic weights (𝔴_*p*′*p*_). Here, the external input (red) combines inputs from background neurons and external inputs given in the complete network model (A). (C) In the network model (A), changing the amplitude of the external input to neuronal populations 1 (black) and 2 (yellow) at *t* = 10 yields increased average activities within the populations and background neurons (blue), triggering synaptic changes. Gray lines indicate single neuron/synapse dynamics. After a brief period, all system variables reach an equilibrium state. This state is matched very well by the theoretical analysis (green lines) considering the abstract model (B). (i) Inputs; (ii) average activities of each population; (iii) average intrapopulation synaptic weights; (iv) average interpopulation synaptic weights. The average input amplitudes are determined by two Ornstein-Uhlenbeck processes with mean ${\tilde{F}}_{1}^{\text{ex}}=0.9$ and ${\tilde{F}}_{2}^{\text{ex}}=0.75$.

To analyze the self-organized formation of memory representations and their functional organization, we consider a plastic recurrent neuronal network model 𝒩 consisting of rate-coded neurons being interconnected via plastic excitatory and static inhibitory connections (Figure 2A). Within the recurrent network are two distinct populations of neurons (*p* ∈{1, 2}; black and yellow dots, respectively) within each the neurons receive the same external input ${\tilde{F}}_{p}^{\text{ex}}$ (red layer *ε*). All remaining neurons are summarized as background neurons ℬ (blue) such that the neuronal network can be described as the interaction of three different neuronal populations.

All excitatory connections within the recurrent layer are plastic regarding the interaction of fast correlation-based synaptic plasticity and slow homeostatic synaptic scaling (Tetzlaff et al., 2011; Tetzlaff, Kolodziejski, Timme, & Wörgötter, 2012). Please note that previous studies indicate that this interaction yields the reliable formation of individual memory representations (Tetzlaff et al., 2013, 2015). The resulting changes in synaptic weightsbetween postsynaptic neuron *i* and presynaptic neuron *j* is thus regulated by

$${\overset{\cdot }{\omega }}_{i,j}=\mu F_{i}F_{j}+\gamma \left({\text{F}}^{\text{T}}-F_{i}\right)\omega_{i,j}^{2}$$ (1)

with neural activities *F*_*i*_ and *F*_*j*_, μ being the timescale of synaptic plasticity, γ the timescale of synaptic scaling, and the target firing rate F^T^ of the homeostatic process.

Thus, an external input to populations 1 and 2 alters neural activities within the corresponding populations and, furthermore, triggers changes in the corresponding synaptic weights (see Figure 2C for an example). In the first phase, all neurons of the network receive a noisy input (Figure 2C, panel i) such that neural activities (panel ii) and synaptic weights (panels iii and iv) are at base level. At *t* = 10, both populations 1 and 2 receive a strong external input (panel i). In more detail, each neuron in a specific population receives an input from 10 input neurons each modeled by its own Ornstein-Uhlenbeck process (grey lines; yellow and black line indicate the average). The mean of these processes is the same for all input neurons transmitting to one population (${\tilde{F}}_{1}^{\text{ex}}=0.9$ for pop. 1 and ${\tilde{F}}_{2}^{\text{ex}}=0.75$ for pop. 2). After a brief transition phase, the system reaches a new equilibrium state. Here, for both populations the intrapopulation synapses are stronger than the average inhibitory synaptic weights ($\tilde{\theta }$; panel iii), indicating the formation of two memory representations. Furthermore, the excitatory synapses connecting both populations are adapted and also become stronger than the average inhibition level (panel iv). This implies that both populations or memory representations are strongly linked with each other; thus, an association has been formed. Therefore, given a certain stimulus, the equilibrium state of the synaptic weights determines the functional organization of the corresponding memory representations.

### Memory Representation and Functional Organization

As the impact of single synapses on the overall network dynamics is small, we will consider in the following the equilibrium states of the average synaptic weights of inter- and intrapopulation synapses (indicated by 〈*x*〉). Thus, these synaptic states determine whether a neuronal population is a memory representation, and how several of these representations are functionally organized (discrimination, sequence, or association).

**Figure 3.**
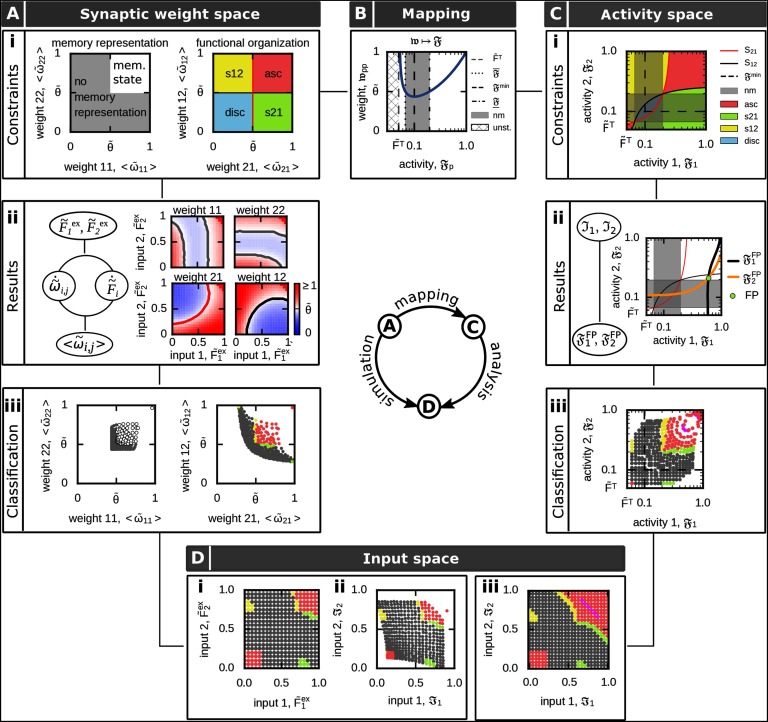
Definition and analysis of the input-dependent formation of functional organizations (FO) between two neuronal populations. For details see main text and Table 1. (Ai) The different FOs are defined based on the average synaptic weights. (Aii) Solving numerically the complete network dynamics, which is of a n^2^-order dimension, for different external inputs (left) yields average excitatory synaptic weights (lines indicate constraints as in Ai). (Aiii) These average synaptic weights (Aii) can be analyzed regarding the weight-dependent conditions of FOs (Ai). Color code of dots as areas in Ai. (B) Considering the dependency of the intrapopulation synaptic weight (𝔴_*pp*_, blue curve; Equation 9) on its respective activity (𝔉_*p*_) in equilibrium enables the mapping of the weight-dependent memory conditions on the 2*d*-activity space. Grey space: no memory representation; white space: memory representation. (Ci) Conditions for the different FOs of two memories in the mapped 𝔉_1_ − 𝔉_2_ − activity space of the neuronal populations (Equations 12–17). (Cii) Within this 2*d*-space one can calculate the fixed point of the population activities 𝔉_1_, 𝔉_2_ by the intersection of the equations 𝔉_1_^FP^ and 𝔉_2_^FP^ given an input stimulation ℑ_1_, ℑ_2_ (here, ℑ_1_ = 0.95, ℑ_2_ = 0.4). (Ciii) By comparing the resulting fixed point from (Cii) with the FO-conditions (Ci), we can obtain the respective FO. (D) Given the results in the weight (A) or activity space (C), we can assess for each input case the resulting FO. Used parameters: $\tilde{\theta }=0.5$, ${\tilde{\text{F}}}^{\text{T}}=0.05$, n_*ϵ*_ = 20.

Therefore, as long as the average recurrent or intrapopulation excitatory synaptic weight $\langle {\tilde{\omega }}_{pp}\rangle$ of neuronal population *p* is weaker than the average inhibitory synaptic strength $\tilde{\theta }$, an external input to the population will lead to an average decrease in population activity ($\tilde{x}$ indicates the normalized variable of *x*; see Methods). Thus, the neuronal population does not serve as a memory representation and the system state is defined as *no memory state* (*nm*; Table 1, Figure 3Ai, left panel, grey area). By contrast, if the average recurrent excitatory synaptic weight is at the equilibrium state above the level of inhibition (Figure 3Ai, left panel, white area), the neuronal population reacts with an increased activity level to an external input and, therefore, it serves as a memory representation (*memory state*). In other words, the neuronal population *p* has to fulfill the following condition to be classified as a memory representation:

$$\tilde{\theta }<\langle {\tilde{\omega }}_{pp}\rangle .$$ (2)

Given that both neuronal populations *p* ∈{1, 2} are a stable memory representation (Figure 3Ai, left panel, white area), they can form different functional organizations (discrimination, sequences, or association). Thereby, the average interpopulation synaptic weights $\langle {\tilde{\omega }}_{p^{\prime }p}\rangle$ ( *p*, *p′* ∈ {1, 2}, *p* ≠ *p′*) define the different functional organizations, dependent on their relation to the average inhibitory synaptic weight strength $\tilde{\theta }$ (Table 1). Thus, for two interconnected memories 1 and 2, we can define four different functional organizations with different weight-dependent conditions (Figure 3Ai, right panel):

▪  *discrimination*: both average interpopulation synaptic weights are weaker than the inhibitory weights (blue, *disc*)
  $$\langle {\tilde{\omega }}_{12}\rangle ,\langle {\tilde{\omega }}_{21}\rangle <\tilde{\theta };$$ (3)
▪  *sequence 21*: average interpopulation synaptic weight from memory 1 to memory 2 is stronger than inhibitory weights, while the interpopulation synaptic weight from 2 to 1 is weaker (green, *s21*)
  $$\langle {\tilde{\omega }}_{12}\rangle <\tilde{\theta }<\langle {\tilde{\omega }}_{21}\rangle ;$$ (4)
▪  *sequence 12*: interpopulation synaptic weight from memory 1 to memory 2 is weaker than the inhibitory weights, while the interpopulation synaptic weight from 2 to 1 is stronger (yellow, *s12*)
  $$\langle {\tilde{\omega }}_{21}\rangle <\tilde{\theta }<\langle {\tilde{\omega }}_{12}\rangle ;$$ (5)
▪  *association*: both average interpopulation synaptic weights are stronger than the average inhibitory synaptic weight (red, *asc*)
  $$\tilde{\theta }<\langle {\tilde{\omega }}_{12}\rangle ,\langle {\tilde{\omega }}_{21}\rangle .$$ (6)

#### Full-network analysis.

Assessing under which input condition the plastic neuronal network is able to form memory representations and diverse functional organizations, the whole set of differential equations, which represents a mathematical problem of the order of *n*^2^ (with *n* neurons), has to be solved numerically for each input condition (${\tilde{\text{F}}}_{\text{1}}^{\text{ex}}$, ${\tilde{\text{F}}}_{\text{2}}^{\text{ex}}$). Thereby, each simulation runs until the system reaches its equilibrium state. In this equilibrium state, excitatory synaptic weights are analyzed and compared with the inhibitory synaptic weights (Figure 3Aii, right panels) enabling a classification according to the functional organizations (Figure 3Aiii). This classification can be mapped to the inputs providing the resulting functional organization dependent on the specific external inputs (Figure 3Di,ii). Note, for better comparison with the population model (see the next section), the results (Figure 3Di) are mapped to the population input space defined below (Figure 3Dii, Equation 7). The whole analysis is computationally expensive and, furthermore, it does not provide additional insights into the relation between the synaptic dynamics and the ability to form diverse functional organizations. Thus, in the following, we provide a different approach to solve this complex, high-dimensional mathematical problem.

#### Population model at equilibrium.

To reduce the complexity of the system, in the following, we derive a method that directly calculates the mean state variables of the memory-related neuronal populations *p* ∈ {1, 2} at equilibrium (Figure 2B). For this we combine the inputs a population receives from the external layer ε (${\tilde{F}}_{p}^{\text{ex}}\;{\tilde{\omega }}^{ex}$) with the inputs from the background neurons in ℬ (${\tilde{F}}_{B}\;{\tilde{\omega }}_{p,B}$) to

$$I_{p}={\tilde{F}}_{p}^{\text{ex}}\;{\tilde{\omega }}^{ex}+\frac{n_{B}}{n_{p}^{\text{ex}}}{\tilde{F}}_{B}\;{\tilde{\omega }}_{p,B}$$ (7)

with *n*_ℬ_ being the number of neurons belonging to the background population ℬ, and *n*_*p*_^ex^ being the number of input neurons. Please note that we use Equation 7 only for transferring the results of the full network simulations to the input space ℑ_1_, ℑ_2_ (Figure 3Dii) to enable comparison with the results from the population model (Figure 3Diii). For the population model derived in the following, we directly consider different levels of ℑ_1_ and ℑ_2_.

Given the input stimulation ℑ_*p*_, we consider that the firing rate ${\tilde{F}}_{i}$ of each neuron *i* ∈ *p* of a population *p* is close to the mean firing rate of this particular population $\langle {\tilde{F}}_{p}\rangle$. By this, we receive the mean neuronal activity of population *p* at equilibrium $F_{p}\approx \langle {\tilde{F}}_{p}\rangle \approx {\tilde{F}}_{i}$ (Equation 38). With the equilibrium activities of the neuronal populations *p* and *p′*, in turn, we can calculate the respective equilibrium synaptic weights 𝔴_*p*′p_ from population *p* to population *p′* (Equation 39):

$$w_{p\prime p}=\sqrt{\frac{F_{p}F_{p\prime }(1-{\tilde{\text{F}}}^{\text{T}})}{F_{p\prime }-{\tilde{\text{F}}}^{\text{T}}}}$$ (8)

and the equilibrium synaptic weights of each population *p* itself

$$w_{pp}=\sqrt{\frac{F_{p}^{2}(1-{\tilde{\text{F}}}^{\text{T}})}{F_{p}-{\tilde{\text{F}}}^{\text{T}}}}.$$ (9)

#### Activity-dependent constraints of memory representation and functional organization.

Given the relation between average population activities and synaptic weights in equilibrium (Equations 8 and 9), next, we map the weight-dependent conditions for memory representations (Equation 2) and functional organizations (Equations 3–6) onto the average population activities (Figure 3B). Thus, the fixed point or equilibrium equation of the synaptic dynamics (Equation 9; blue curve in Figure 3B) yields two activity-dependent conditions of a neuronal population *p* to become a memory representation:

$$F_{p}<\frac{{\tilde{\theta }}^{2}-\tilde{\theta }\sqrt{D}}{2(1-{\tilde{\text{F}}}^{\text{T}})},$$ (10)

$$F_{p}>\frac{{\tilde{\theta }}^{2}+\tilde{\theta }\sqrt{D}}{2(1-{\tilde{\text{F}}}^{\text{T}})}$$ (11)

with $D={\tilde{\theta }}^{2}-4{\tilde{\text{F}}}^{\text{T}}(1-{\tilde{\text{F}}}^{\text{T}})$. Thus, we can define two open intervals (Figure 3B, white regimes) for the population activity leading to a representation of the respective memory by:

$$\begin{array}{ll} {\text{lower}}_{F}:=\left({\tilde{\text{F}}}^{\text{T}},\bar{F}\right) & \;\text{with}\bar{F}:=\frac{{\tilde{\theta }}^{2}-\tilde{\theta }\sqrt{D}}{2(1-{\tilde{\text{F}}}^{\text{T}})}, \end{array}$$ (12)

and

$$\begin{array}{ll} {\text{upper}}_{F}:=\left(\underline{F},1\right) & \;\text{with }\underline{F}:=\frac{{\tilde{\theta }}^{2}+\tilde{\theta }\sqrt{D}}{2(1-{\tilde{\text{F}}}^{\text{T}})}. \end{array}$$ (13)

Note that below the target firing rate ${\tilde{\text{F}}}^{\text{T}}$the interaction of synaptic plasticity and scaling does not have a fixed point (Figure 3B, hatched regime; Equation 1). Furthermore, in the regime $\bar{F}<F_{p}<\underline{F}$ no proper memory representation can be formed (equivalent to $w_{pp}\leq \tilde{\theta }$). This activity regime is defined as the *no memory* state $\text{nm}:=[\bar{F},\underline{F}]$ (Figure 3B, grey regime) with size $\left|\text{nm}|=\tilde{\theta }\sqrt{D}/(1-{\tilde{\text{F}}}^{\text{T}}\right)$.

As we consider the interaction of two interconnected neuronal populations 1 and 2, we receive four distinct activity regimes enabling the formation of two memory representations (Figure 3Ci). These regimes are defined by all possible combinations of lower_𝔉_ and upper_𝔉_ in both dimensions of 𝔉_1_ and 𝔉_2_. In other words, these activity regimes are separated by the no memory phase (nm) in both dimensions (Figure 3Ci, grey regimes).

Similarly, with Equation 8, we can map the diverse conditions of the functional organizations (Equations 3–6) onto different activity-dependent conditions. In general, the condition $w_{p\prime p}<\tilde{\theta }$ becomes

$$F_{p}<\frac{{\tilde{\theta }}^{2}}{1-{\tilde{\text{F}}}^{\text{T}}}\left(1-\frac{{\tilde{\text{F}}}^{\text{T}}}{F_{p^{\prime }}}\right)$$ (14)

and $w_{p\prime p}>\tilde{\theta }$ to

$$F_{p}>\frac{{\tilde{\theta }}^{2}}{1-{\tilde{\text{F}}}^{\text{T}}}\left(1-\frac{{\tilde{\text{F}}}^{\text{T}}}{F_{p^{\prime }}}\right)\;.$$ (15)

To distinguish between both cases (which determines the functional organization between two memories 1 and 2), we define two separatrices:

$$S_{21}:=F_{1}=\frac{{\tilde{\theta }}^{2}}{1-{\tilde{\text{F}}}^{\text{T}}}\left(1-\frac{{\tilde{\text{F}}}^{\text{T}}}{F_{2}}\right),$$ (16)

$$S_{12}:=F_{2}=\frac{{\tilde{\theta }}^{2}}{1-{\tilde{\text{F}}}^{\text{T}}}\left(1-\frac{{\tilde{\text{F}}}^{\text{T}}}{F_{1}}\right).$$ (17)

Thus, *S*_21_ represents $w_{21}=\tilde{\theta }$ in the activity-space (Figure 3Ai, Ci, red curves) while *S*_12_ represents $w_{12}=\tilde{\theta }$ (Figure 3Ai, Ci, black curves). The relation of each activity level according to its separatrix determines the actual functional organization:

*Discrimination.*  When both activities 𝔉_1_ and 𝔉_2_ are below the respective separatrix *S*_21_ and *S*_12_ (Figure 3Ci, blue area), the system is in an discriminatory functional organization.
*Sequence.*  The system establishes a sequence from memory 1 to memory 2, when the activity 𝔉_1_ is above the corresponding separatrix *S*_21_ while the activity 𝔉_2_ stays below separatrix *S*_12_ (Figure 3Ci, green area, s21) and vice versa for a sequence from memory 2 to memory 1 (yellow area, s12).
*Association.*  Both memories are organized in an associational entity when both neuronal activities 𝔉_1_ and 𝔉_2_ are above their respective separatrix (Figure 3Ci, red area).

#### Functional organizations in activity-space.

To obtain which functional organization the system forms for a given external input, we have to calculate the input-dependent average population activities 𝔉_1_ and 𝔉_2_ in the equilibrium state. For this, for each pair of inputs ℑ_1_ and ℑ_2_, we derive the fixed point conditions for both populations (Equation 43) dependent on the activity of the other population (Figure 3Cii; 𝔉_1_^FP^(𝔉_2_), black curve; 𝔉_2_^FP^(𝔉_1_), yellow curve). The intersection between both fixed point conditions (𝔉_1_^FP^ = 𝔉_2_^FP^) determines the fixed point of the whole system (green dot). The relation of the corresponding activities 𝔉_1_ and 𝔉_2_ of this intersection to the separatrices determines the functional organization (Figure 3Ciii). This can be expressed in the input space (Figure 3Diii). Thus, the interaction of synaptic plasticity and scaling enables the formation of sequences in both directions and associations. Furthermore, there is a regime of input values in which no memory representation is formed.

Comparing the analytical results from the population model (Figure 3Diii) with the results from the full network analysis (Figure 3Dii) indicates that the population model matches the full network quite well. Especially, the inherent property of a system to form different functional organizations is precisely predicted by the population model. Remarkably, already the mapping of the weight-dependent conditions on the activity space (Figure 3Ci) provides sufficient information to assess the possible organizations of memories for a given system (not requiring the evaluation of the system’s fixed points).

#### Synaptic-plasticity-induced formation of associations.

**Figure 4.**
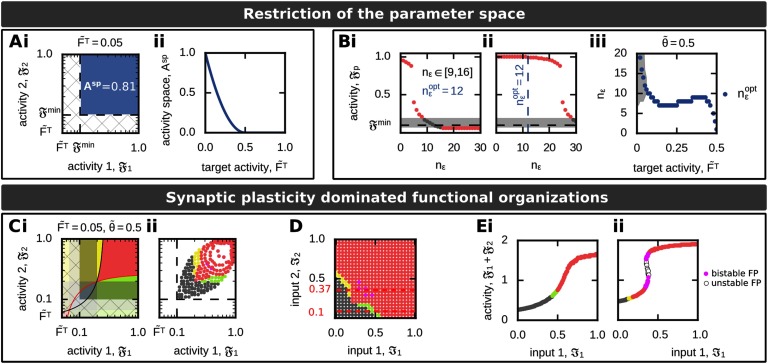
Synaptic plasticity dominated functional organization (FO) of two interconnected memories. (A, B) The regime A^SP^, in which synaptic plasticity dominates the synaptic dynamics, depends on the target firing rate ${\tilde{\text{F}}}^{\text{T}}$ (A) and inflexion point ϵ = n_ϵ_*u*^max^ (B). (A) The area of the 𝔉_1_ − 𝔉_2_ − activity phase space (Ai, blue space) leading to synaptic plasticity dominated FOs decreases with increasing target firing rate ${\tilde{\text{F}}}^{\text{T}}$ (Aii). (B) The inflexion point (measured in n_ϵ_) determines the activity-input mapping such that for the same input different activities and, thus, different FOs are realized. If the inflexion point equals n_ϵ_^opt^, 𝔉_1_ ≈ 𝔉^min^. (Bi): Input: ℑ_1_ = ℑ_2_ = 0. (Bii): Input: ℑ_1_ = ℑ_2_ = 1. (Biii) The value of n_ϵ_^opt^ (blue) depends on the target firing rate ${\tilde{\text{F}}}^{\text{T}}$. The grey area specifies all n_ϵ_ that yield to the no-memory state. (C–E) One example of synaptic plasticity dominated formation of FOs. Used parameters: ${\tilde{\text{F}}}^{\text{T}}=0.05$, n_ϵ_ = 12. (C) Although the system implies regimes of scaling-dominated synaptic dynamics (hatched area; i), the activity-input mapping excludes that the system can reach these by external inputs (ii). (D) The resulting ℑ_1_ − ℑ_2_ − input phase space, color-coded according to the respective FOs, shows that associations can only be formed for stronger inputs (compare to Figure 3Diii). (E) The sum of both population activities (𝔉_1_ + 𝔉_2_) for a fixed input ℑ_2_ shows for some cases the existence of two equilibrium states encoding associations (pink). (Ei): Input: ℑ_2_ = 0.1. (Eii): Input: ℑ_2_ = 0.37. Color code see Table 1.

Both analysis methods (Figure 3A and C) indicate that the interaction of synaptic plasticity and scaling enables the formation of sequences and associations between two memory representations (Figure 3D). Interestingly, for very low external input stimulations ℑ_1_ and ℑ_2_, the system forms an association. This is mainly due to the quadratic weight-dependency of synaptic scaling (Equation 1) such that for low population activities synaptic scaling dominates the synaptic dynamics and drives the synaptic weights to high values (up-scaling). This is in contrast to the synaptic-plasticity-and-memory hypothesis (Martin et al., 2000; Hebb, 1949), which states that the processes of correlation-based synaptic plasticity dominates learning. Along this line, synaptic scaling should mainly regulate the synaptic dynamics in an homeostatic manner (Turrigiano & Nelson, 2004). In other words, the synaptic weights should mainly increase with increasing neuronal activities. To determine the regime in which this behavior is present, we consider the activity level $\left(F^{\text{min}}=2{\tilde{\text{F}}}^{\text{T}}\right)$ yielding the local minimum of the synaptic weight function (Equation 9; Figure 3B). Below 𝔉_min_ ($F_{p}\in ({\tilde{\text{F}}}^{\text{T}},F^{\text{min}})$), the dynamics are dominated by synaptic scaling and should be avoided. Thus, the plausible activity regime is in the

$$[F^{\text{min}},1]\times [F^{\text{min}},1]-\text{activity space}$$ (18)

(Figure 4Ai, blue space) as in this space the synaptic weight dynamics are dominated by correlation-based synaptic plasticity. This regime exists as long as the target activity ${\tilde{\text{F}}}^{\text{T}}$ is below 0.5 $\left({\text{A}}^{\text{SP}}={\left(1-2\;{\tilde{\text{F}}}^{\text{T}}\right)}^{2}\right)$ restricting the target activity parameter to ${\tilde{\text{F}}}^{\text{T}}\in (0,0.5)$ (Figure 4Aii).

**Theorem 1** The activity regime lower_𝔉_ (Equation 12), which enables a proper formation of memory representations, is not part of the correlation-based dominated activity space (18) for synaptic plasticity.

*Proof.* Assume that the upper bound $\bar{F}$ of lower_𝔉_ is smaller than the lower bound 𝔉^min^ for the synaptic plasticity dominated activity regime (Equation 18). It follows that the condition ${\tilde{\text{F}}}^{\text{T}}(1-{\tilde{\text{F}}}^{\text{T}})>0$ is true for ${\tilde{\text{F}}}^{\text{T}}\in (0,0.5)$ and by this lower_𝔉_ ∉ A^sp^.

Thus, to assure that the activity regime lower_𝔉_ cannot be reached by the system, we have to change the mapping between neuronal activity and inputs such that no reasonable input pair ℑ_1_, ℑ_2_ yields population activities within lower_𝔉_. This activity-input mapping is mainly determined by the inflexion point ϵ of the activity function (Equations 33 and 43). Here, we specify the inflexion point in units of n_ϵ_ (ϵ =n_ϵ_*u*^max^) with n_ϵ_ being the number of maximally active presynaptic neurons (with maximally strong synapses; see Methods). First, we analyze the resulting population activities 𝔉_*p*_ and corresponding functional organizations for different n_ϵ_ given no external inputs (ℑ_1_ = ℑ_2_= 0; Figure 4Bi). For n_ϵ_ > 12, the population activities are below 𝔉^min^, which triggers up-scaling yielding a scaling-induced formation of an association. Please note that the system analyzed beforehand (Figure 3) has n_ϵ_ = 20. For n_ϵ_ < 9, neurons are too easy to excite such that activities are independent of the input nearby the maximum, yielding the functional organization of association. For 9 ≤ n _ϵ_ ≤ 16, the system is in the no- memory state. Thus, to prevent the input-*in*dependent association of two interconnected neuronal populations, we consider the inflexion point to be in the regime 9 ≤ n_ϵ_ ≤ 16. Thereby, n_ϵ_^opt^ = 12 yields activities nearby the lower minimum activity level 𝔉^min^ defined above. The same analysis for maximal external input stimulation (ℑ_1_ = ℑ_2_ = 1; Figure 4Bii) shows that for n_ϵ_^opt^ = 12 the system can nearly reach its maximal firing rate of 𝔉_*p*_ = 1 such that the whole activity space [𝔉^min^, 1] can be reached by the system. Please note that for n_ϵ_ > 24, the system cannot reach high activity levels, and for n_ϵ_ > 27 the system is not able to form memory representations, although it is maximally stimulated by the external input. Furthermore, as can be expected from Figure 4A, the value of n_ϵ_^opt^ depends on the target firing rate ${\tilde{\text{F}}}^{\text{T}}$ (Figure 4Biii).

In the following (Figure 4C–E), we will consider n_ϵ_ = 12 and $\tilde{{\text{F}}^{\text{T}}}=0.05$, which implies that an association is only be formed by synaptic dynamics dominated by correlation-based synaptic plasticity. The activity regime yielding scaling-dominated learning (hatched area in Figure 4Ci) is theoretically possible; however, the adapted activity-input mapping assures that this regime cannot be reached for given external inputs (Figure 4Cii and D). In the resulting system, low inputs ℑ_1_, ℑ_2_ lead to a no-memory state (grey), while in a small regime sequences are formed (yellow and green). Thereby, the sequence is formed from the population receiving a stronger input to the population receiving the weaker input. If both inputs are strong, an association between the memory representations is being built (red). Note that there is a small bimodal regime with two long-term equilibrium states both being an association (pink; see two exemplary cross sections in Figure 4E).

#### Parameter-dependency of functional organizations.

After optimizing the activity-input mapping by n_ϵ_ such that the formation of diverse functional organizations is dominated by synaptic plasticity, in the following, we will analyze which kind of functional organizations can be formed by the system dependent on the different system parameters. Thereby, we will focus on the target activity ${\tilde{\text{F}}}^{\text{T}}$ and the average level of inhibition $\tilde{\theta }$.

**Figure 5.**
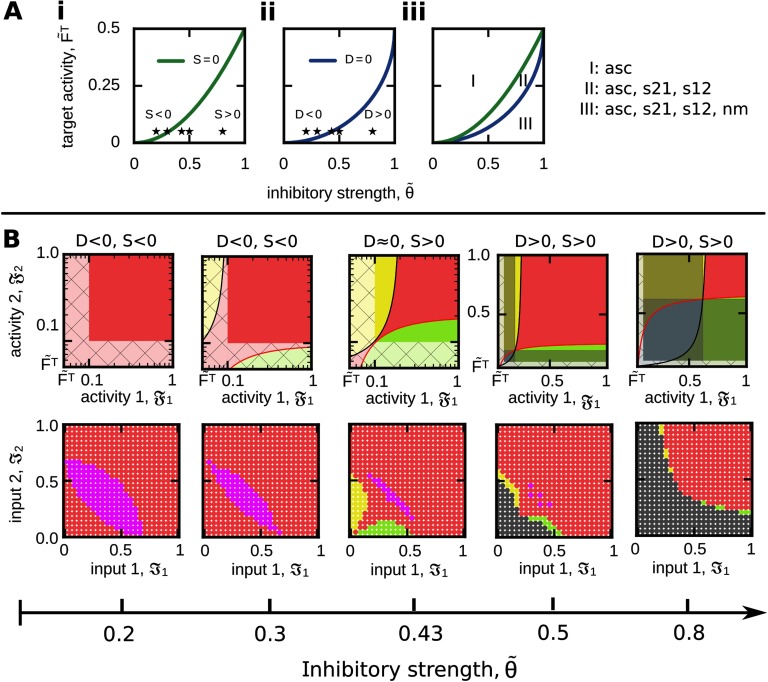
Quantification of the system ability to form different FOs dependent on the parameters ${\tilde{\text{F}}}^{\text{T}}$ and $\tilde{\theta }$. (A) The measure *S* (i; green) indicates whether sequences can be formed, while the measure *D* (ii; blue) specifies the existence of no-memory states. (iii) Both measures together separate the ${\tilde{\text{F}}}^{\text{T}}-\tilde{\theta }-$parameter phase space into three distinct regimes. Please see main text for details. (B) For a constant target activity (${\tilde{\text{F}}}^{\text{T}}=0.05$), we show several examples of resulting functional organizations for different values of inhibition $\tilde{\theta }$ (asterisks in (A)) in activity (top row) and input space (bottom). Color code see Table 1.

In general, as the no-memory state implies that neuronal populations can exist that do not encode information (or have “forgotten” this information), this state has a large influence on the overall system properties. As described above, the size of this state is given by $\left|\text{nm}|\;=\tilde{\theta }\sqrt{D}/(1-{\tilde{\text{F}}}^{\text{T}}\right)$ with $D={\tilde{\theta }}^{2}-4{\tilde{\text{F}}}^{\text{T}}(1-{\tilde{\text{F}}}^{\text{T}})$. Thus, the discriminant *D* or rather ${\tilde{\text{F}}}^{\text{T}}$ and $\tilde{\theta }$ define whether the no-memory state can exist in a given system (Figure 5). In addition, as the relation of the activity levels to the separatrices (Equations 16 and 17) define which kind of functional organization is present (see above), the separatrices have to be within the synaptic plasticity dominated activity regime (𝔉_*p*_ ∈ (𝔉^min^, 1)) to enable the formation of sequences (*s12*: 𝔉_1_ < *S*_21_, 𝔉_2_ > *S*_12_; *s21*: 𝔉_1_ >, *S*_21_, 𝔉_2_ < *S*_12_) and discrimination (*disc*: 𝔉_1_ < *S*_21_, 𝔉_2_ < *S*_12_). As *S*_12_ (*S*_21_) increase with 𝔉_1_ (𝔉_2_), the maximum difference *S* between the lower activity level of synaptic plasticity dominated dynamics and the separatrix is given for 𝔉_1_ = 1 (𝔉_2_ = 1) such that

$$S=S_{12}[F_{1}=1]-F^{\text{min}}={\tilde{\theta }}^{2}-2\;{\tilde{\text{F}}}^{\text{T}}.$$ (19)

Thus, the ${\tilde{\text{F}}}^{\text{T}}$-$\tilde{\theta }$-dependency of *D* (Figure 5Ai) and *S* (Figure 5Aii) determines the potential of the system to form diverse functional organizations (Figure 5Aiii). Interestingly, there are three functionally different system configurations: for *D* < 0, *S* < 0, the system can only form associations (regime I in Figure 5Aiii; first and second column in Figure 5B). If *D* < 0, *S* > 0, the system can form either associations or sequences (*s12* as well as *s21*; regime II; third column). And if *D* > 0, *S* > 0, associations, sequences, and the no-memory state can be formed and reached by the system (regime III; fourth and fifth column). Thus, this analysis shows that with larger average inhibitory weight $\tilde{\theta }$ and smaller target activity level ${\tilde{\text{F}}}^{\text{T}}$ the system receives a larger repertoire of functional organizations. However, this analysis also shows that the functional organization of discrimination cannot be formed in a long-term manner. Although for large values of inhibition both separatrices intersect; thus, both activity levels could be simultaneously below their corresponding separatrix (see, for instance, blue area in fifth column in Figure 5B), the resulting area of discrimination cannot be reached by any inputs ℑ_1_, ℑ_2_, because in all these cases the neuronal populations cannot serve as memory representations (grey).

**Theorem 2** Correlation-based synaptic plasticity in combination with an activity-dependent postsynaptic synaptic scaling term lacks the formation of functionally unrelated memories (discrimination).

*Proof.* The constraints for a discrimination of two memories can be summarized by two inequations regarding the mean synaptic weights of the neuronal population model:

$$w_{12}<\theta <w_{11}\;\Rightarrow \;F_{1}>F_{2},$$ (20)

and

$$w_{21}<\theta <w_{22}\;\Rightarrow \;F_{1}<F_{2}.$$ (21)

We easily see that the first condition (Equation 20) is in contradiction to the second condition (Equation 21) and, thus, the discrimination of two interconnected memories is excluded.

Thus, a neuronal system with correlation-based synaptic plasticity and a postsynaptic-activity-dependent synaptic scaling is not able to form two excitatory relations in between two memory representations that are weaker than the average inhibition. This analysis reveals that applying such a learning rule globally for the neuronal network dynamics is not sufficient to distinguish the two different processes of memory formation and discrimination. Thus, it seems that this learning rule has to be augmented by at least one additional adaptive process that decouples these two processes.

#### Local Inhibition Enables the Functional Organization of Discrimination

The ability to form a discriminatory relation between memory representations is functionally very important for a neuronal system, as it implies that not all memories, which are anatomically connected with each other, have to be functionally connected with each other. Thus, to overcome the lack of discriminatory functional organizations of memories, we have to “decouple” the discrimination condition from the memory condition (see above).

**Figure 6.**
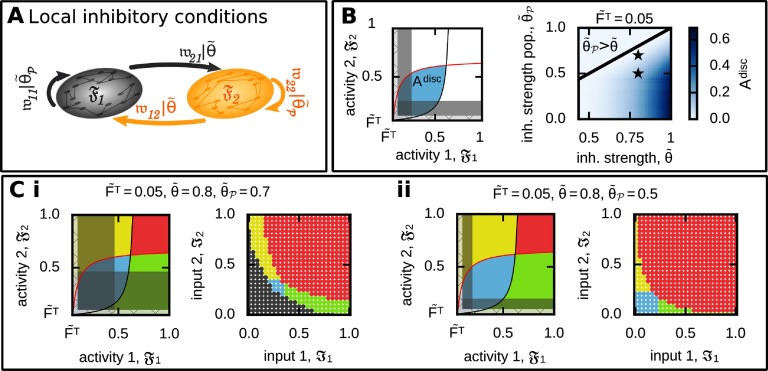
Considering different levels of inhibition level for connections within the neuronal populations compared with all others enables the formation of two discriminated memory representations. (A) We consider a different average inhibitory synaptic weight within the neuronal populations (${\tilde{\theta }}_{p}$) compared with all others ($\tilde{\theta }$). (B) Left: To quantify the effect of different inhibition levels, we calculate the area of discrimination states (A^disc^; blue) not being “covered” by the no-memory states (grey) in the 𝔉_1_-𝔉_2_-activity space. Right: A^disc^ dependency on different relations between ${\tilde{\theta }}_{p}$ and $\tilde{\theta }$. (C) Given a lower level of inhibition within the populations than otherwise provides the neural system the ability to form all functional organizations as indicated here by two examples (asterisked in (B)); (i) $\tilde{\theta }=0.8$, ${\tilde{\theta }}_{p}=0.7$; (ii) $\tilde{\theta }=0.8$, ${\tilde{\theta }}_{p}=0.5$. Color code see Table 1.

For this, we introduce a different inhibitory synaptic weight strength (${\tilde{\theta }}_{P}=\frac{1}{n_{p}^{2}}\sum_{i,j\in p}{\tilde{w}}_{j,i}^{-}$) within the neuronal populations compared with the inhibitory synaptic weight strength for all other connections ($\tilde{\theta }$, Figure 6A). In other words, the parameter $\tilde{\theta }$ is different for the discrimination condition as for the memory condition (which is now ${\tilde{\theta }}_{P}$). To quantify the influence of this new parameter on the potential to form two discriminated memory representations, we calculate the size of the activity space leading to discrimination (Figure 6B, left). In general, if inhibition within the populations is weaker than for all other connections (${\tilde{\theta }}_{P}<\tilde{\theta }$), the system can form memories being in a discrimination (Figure 6B, right). Please note that the other functional organizations are still maintained such that all different types can be obtained (Figure 6C). Interestingly, the state of discrimination is being formed if the inputs presented to both populations are weak. A weak input means that, among others, the probability of occurrence is low, which implies that the chance of both inputs being presented simultaneously is very low (Figure 1). In other words, if both inputs are only accidentally shown simultaneously, the neuronal system should discriminate their memory representations. Vice versa, if the inputs are often shown together (as for high input levels), the system should associate the representations as the interplay between synaptic plasticity, scaling, and inhibition does (Figure 6C).

**Figure 7.**
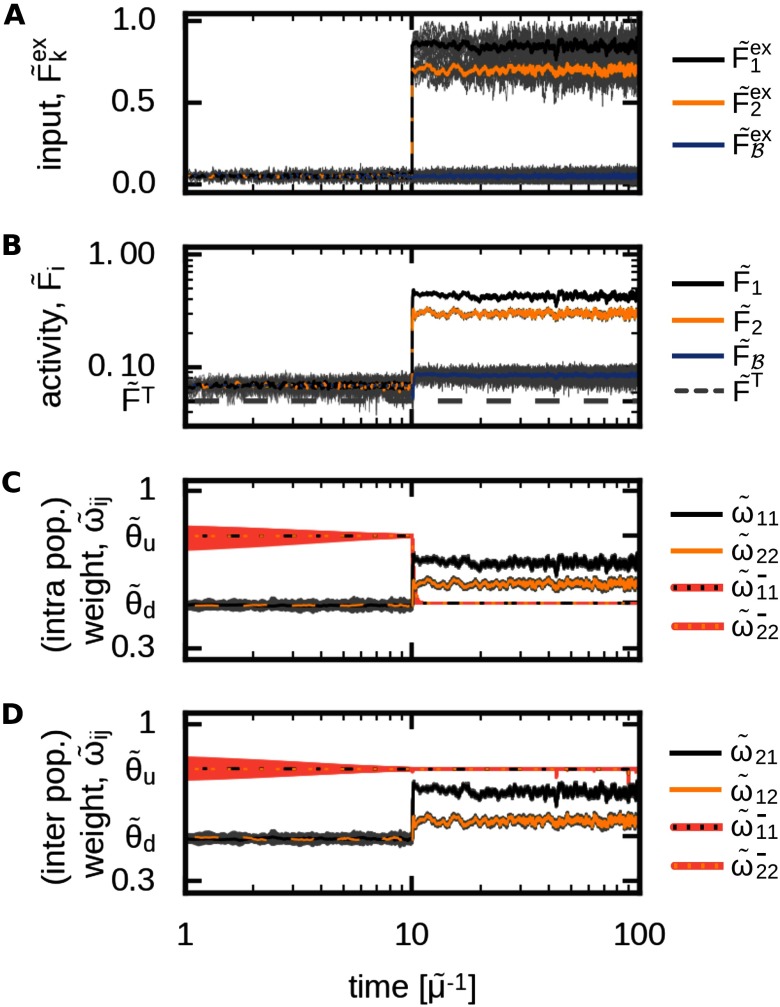
An exemplary inhibitory plasticity rule enables the self-organized formation of a discriminatory functional organization. The development of the input-driven dynamics of the complete neural network (Figure 2) with inhibitory plasticity (Equation 44). (A) The average input amplitudes are determined by two Ornstein-Uhlenbeck processes with mean ${\tilde{\text{F}}}_{1}^{\text{ex}}=0.85$ and ${\tilde{\text{F}}}_{2}^{\text{ex}}=0.7$. (B) Average activities of each population; (C) average intrapopulation excitatory and inhibitory synaptic weights; (D) average interpopulation excitatory and inhibitory synaptic weights.

Although in the analysis above we predefined different levels of inhibition, these different levels can also be obtained by the system in a self-organized manner by considering inhibitory synaptic plasticity (see Figure 7 for an example of discrimination). Similar to excitatory synaptic plasticity, the here-used example of inhibitory synaptic plasticity depends on the correlation of pre- and postsynaptic firing. In addition, the inhibitory synaptic plasticity rule is multiplied by two additional constrains. First, a minimum activity level θ_*F*_ for the pre- and postsynaptic firing rates introduces a threshold for inhibitory synaptic plasticity to occur

$${\overset{\cdot }{\tilde{\omega }}}_{j,i}^{-}\propto {\tilde{F}}_{i}{\tilde{F}}_{j}\text{H}(\sum F-\theta_{F}),$$ (22)

with ${\overset{\cdot }{\tilde{\omega }}}_{j,i}^{-}$ being the strength of the inhibitory synapse connecting the presynaptic neuron *i* with the postsynaptic neuron *j*, $\sum F:={\tilde{F}}_{i}+{\tilde{F}}_{j}$, and H being the Heaviside step function. Second, the difference in the pre- and postsynaptic firing rates ($\Delta F:=|{\tilde{F}}_{i}-{\tilde{F}}_{j}|$) provides an abstract measure for the noncorrelation of firing due to large deviations in their firing rates.

$${\overset{\cdot }{\tilde{\omega }}}_{j,i}^{-}\propto {\tilde{F}}_{i}{\tilde{F}}_{j}\text{H}(\delta F-\Delta F).$$ (23)

Here, *δF* describes a tolerance range for such a variation in the firing rates.

According to these conditions, the inhibitory synaptic weights converge either to

▪  an up-state (θ_*u*_), if the sum of neuronal activities is smaller than its threshold (∑*F* < θ_F_) **and/or** the difference in the pre- and postsynaptic activities is above its tolerance range (Δ*F* > *δF*), or
▪  a down-state (θ_*d*_), if the sum of neuronal activities is larger than its threshold (∑*F* > θ_F_) **and** the difference in the pre- and postsynaptic activities is smaller than its tolerance range (Δ*F* < *δF*).

This type of inhibitory synaptic plasticity (Equation 44) together with plastic excitatory synapses governed by the interaction of correlation-based synaptic and homeostatic plasticity enables the reliable formation of memory representations and, in addition, provides the system the ability to form all basic functional organizations. In other words, our analyses indicate that a self-organized neural network can form all types of functional organizations if the interaction of synaptic plasticity and scaling are complemented by further adaptive processes.

#### Generalization of the Interaction Between Multiple Memory Representations

**Figure 8.**
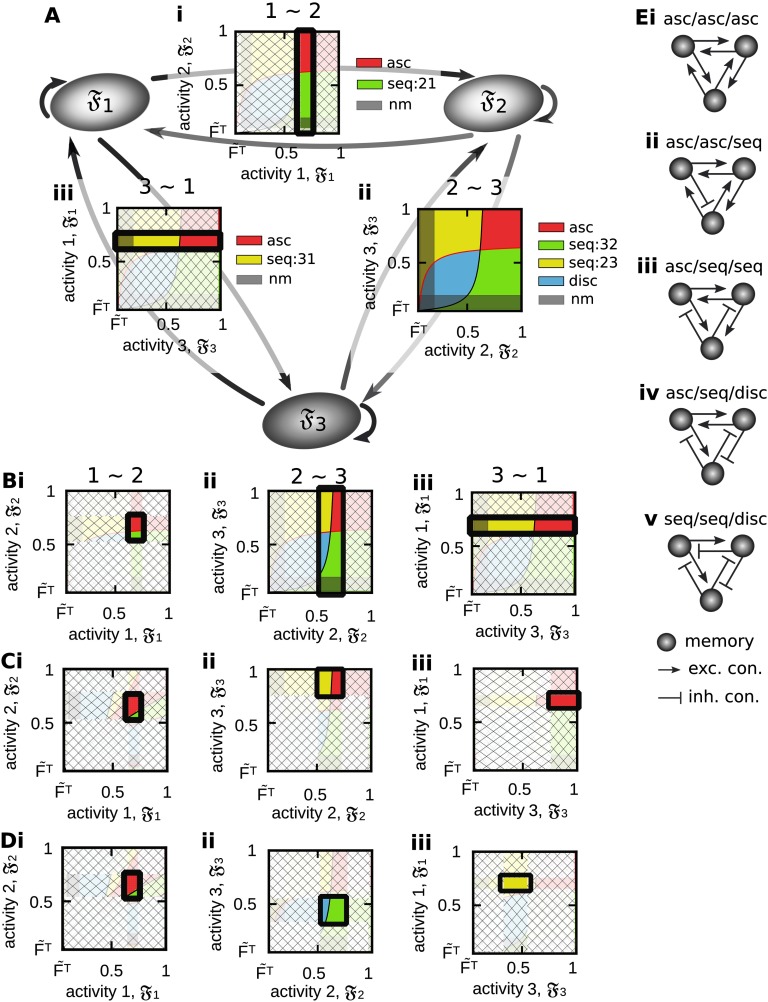
By considering the results from analyzing the relations between two memory representations, we can infer possible functional organizations between three neuronal populations. (A) If we assume that the external input can constrain the activity of a neuronal population to a specific value, constraining for instance the average activity of population 1 to 𝔉_1_ ∈ [0.65, 0.8] restricts its relation to populations 2 (i) and 3 (iii), while the relation between population 2 and 3 is not limited (ii; compare to Figure 6Cii, left). (B) If the activity of population 2 is also constrained (𝔉_2_ ∈ [0.5, 0.75]), the relation between population 2 and 3 becomes also limited (ii). (C, D) If the activity of population 3 is also limited to a certain interval (C: 𝔉_3_ ∈ [0.75, 1]; D: 𝔉_3_ ∈ [0.3, 0.55]), the functional organizations between all three populations are determined. (E) Different possible functional organizations for the examples shown in C and D. Color code see Table 1.

The analyses shown before are focused on the functional organization between two memory representations. However, given the results from these analyses, we can infer which types of functional organizations can be formed between three memory representations (Figure 8A). For this, we have to consider the space of possible functional organizations for different levels of activities 𝔉_*p*_ and 𝔉_*p*′_, *p*, *p′*∈{1, 2, 3} between two neuronal populations (e.g., resulting from the interaction between synaptic plasticity and scaling and different levels of inhibition as shown in Figure 6Cii, left). This space implies that if two populations are in a specific functional organization, the activity levels of the corresponding populations are determined to specific intervals that in turn, constrain the functional organization between these populations and a third one. In other words, if we constrain the activity level of population 1 by the external input onto, without loss of generality, the interval 𝔉_1_ ∈ [0.65, 0.8], the spaces of functional organizations between population 1 and 2 (Figure 8Ai) and between population 1 and 3 (Figure 8Aii) are limited onto specific regimes such that only a subset of functional organizations can be realized. As long as we do not constrain the activity levels of population 2 and 3, these two populations are able to form all types of functional organizations (Figure 8Aii). If we also constrain the activity level of, for example, the second population (𝔉_2_ ∈ [0.5, 0.75]), the functional organization between population 1 and 2 is basically specified (association) and the space of functional organizations between population 2 and 3 is limited. Now, if also the activity level of the third population is constrained (𝔉_3_ ∈ [0.75, 1] in Figure 8C and 𝔉_3_ ∈ [0.3, 0.55] in D), all three possible interactions between the three memory representations are defined. By the above described procedure, we can infer which functional organizations between three memory representations can be reliably formed (see Figure 8E for examples given in C and D). Numerical simulations are required to confirm these results. However, we expect that, by applying procedures as described above, the here-developed framework can be extended to investigate the ability of diverse plasticity mechanisms to form different types of webs of memories.

## DISCUSSION

### General Framework

In the present work, we have developed a mathematical framework to investigate the ability of adaptive neural networks to form in a dynamic, input-dependent manner diverse functional organizations of interconnected memories. In contrast to previous studies focusing only on a subset of possible functional organizations (Chenkov et al., 2017; Tully et al., 2016; Griniasty et al., 1993; Abbott & Blum, 1996; Leibold & Kempter, 2006; Herrera-Aguilar, Larralde, & Aldana, 2012), we consider here all possible organizations between two memory representations. Thereby, we define the functional organizations dependent on the relation between the excitatory and inhibitory synaptic weights of the neuronal network. By introducing a population description, we are able to transfer the resulting high-dimensional problem to a low-dimensional problem considering average synaptic weights and activities of the neuronal populations involved. In addition, by considering the long-term equilibrium dynamics, we could further reduce the system complexity with the input stimulation being a system parameter. Finally, we could map the resulting dynamics onto the two-dimensional activity space which is sufficient to solve this complex problem of memory interactions (Figure 3). Thus, we gain an easily accessible understanding of the possible states the system can reach as well as of the underlying principles arising from the considered plasticity mechanisms and their limitations. Given the generality of the complete framework, it can be commonly used to investigate the effect of diverse plasticity mechanisms on the formation and interaction between memory representations.

### Analysis of the Interplay Between Synaptic Plasticity and Synaptic Scaling

Given this general mathematical framework, we analyzed the effect of the interplay of correlation-based synaptic plasticity with homeostatic synaptic scaling on the formation of functional organizations of memory. This type of interplay is a quite general formulation of synaptic dynamics (Tetzlaff et al., 2011; Abbott & Nelson, 2000), which is sufficient to form individual memory representations (Tetzlaff et al., 2013, 2015). We have shown that these types of mechanisms provide a neural network the ability to form several types of functional organization of memory representations such as sequences and associations (Figures 4 and5). Furthermore, our method shows that correlation-based plasticity with scaling does not enable the formation of two stable memory representations being in a discriminated state.

This shortcoming is due to the purely correlation-based formulation of synaptic plasticity and, by this, mathematically couples the condition for memory formation with the condition of discrimination. Interestingly, the correlation-independent dynamics triggered by synaptic scaling are not sufficient to decouple the conditions. However, these dynamics enable the formation of sequences providing a further functional role of synaptic scaling besides synaptic stabilization (Tetzlaff et al., 2011; Zenke et al., 2013; Zenke, Gerstner, & Ganguli, 2017) and homeostatic regulation of neuronal activities (Turrigiano & Nelson, 2004; Abbott & Nelson, 2000).

On the basis of our results, we expect that similar mathematical models of synaptic dynamics, which consist of correlation-based plasticity and a homeostatic term dependent on the postsynaptic activity level (e.g., Oja’s rule (Oja, 1982) or BCM rule (Bienenstock, Cooper, & Munro, 1982)) are also not able to form memory representations in a discriminated state. Thus, a further factor determining the synaptic dynamics of the network is required to enable the functional organization of discrimination.

### Local Variations of Inhibition

We have shown that local variations in the level of inhibition could serve as such a factor enabling the discrimination between memory representations and other functional organizations (Figure 6). Thereby, the average inhibitory synaptic strength within a memory representation has to be weaker than all other inhibitory synaptic weights. This is in contrast to the idea of an inhibition, which balances the strong excitation within interconnected groups of neurons (Litwin-Kumar & Doiron, 2014; Vogels, Sprekeler, Zenke, Clopath, & Gerstner, 2011). However, despite the local differences in the balance of inhibition and excitation, the network-wide levels of excitation and inhibition can still be in a balanced state (van Vreeswijk & Sompolinsky, 1998; Denève & Machens, 2016). Furthermore, this type of inhibitory weight structure could emerge from an anti-Hebbian-like inhibitory plasticity rule as discovered in the memory-related hippocampus (Woodin, Ganguly, & Poo, 2003).

### Possible Extensions of Synaptic Dynamics

Besides inhibition, other mechanisms could be the additional factor yielding all functional organizations. For instance, spike-timing-dependent plasticity (STDP; Gerstner, Kempter, van Hemmen, & Wagner, 1996; Bi & Poo, 1998; Markram, Gerstner, & Sjöström, 2011) adapts the synaptic weights according to the correlation of pre- and postsynaptic spiking dynamics. By considering detailed models of this mechanism (van Rossum, Bi, & Turrigiano, 2000; Song, Miller, & Abbott, 2000; Shouval, Bear, & Cooper, 2002; Graupner & Brunel, 2012), the influence of time-dependent properties of the stimuli, as correlations, on the formation of functional organizations of multiple memory representations can be investigated. Previous studies already indicated that STDP together with other plasticity mechanisms can reliably form memory representations (Litwin-Kumar & Doiron, 2014; Zenke et al., 2015); however, the interaction between such memory representations and the ability to form diverse functional organizations given the mechanism of STDP remains unclear. For a more detailed understanding, the theoretical framework presented in this study could be used. For this, the framework has to be adapted such that it takes the dynamics of STDP and spikes into account. This requires differential equations describing the dynamics of populations of neurons of a certain size, given synaptic plasticity. In a recent study (Schwalger, Deger, & Gerstner, 2017), the authors derive a mathematical model of populations of a certain number of spiking neurons, which also considers the dynamics of short-term synaptic plasticity (Schmutz, Gerstner, & Schwalger, 2018). However, a mathematical model describing the dynamics of a population of spiking neurons (of fixed size) with STDP, which would be essential to extend the here-presented framework by time-dependent properties of stimuli, is still missing.

This methodical gap could be at least partially circumvented by extending the rate-dependent synaptic plasticity model. For instance, spike-timing-dependent triggered LTD (Bi & Poo, 1998; van Rossum et al., 2000), in contrast to firing rate-dependent LTD (Bienenstock et al., 1982; Sjöström, Turrigiano, & Nelson, 2001; Malenka & Bear, 2004), could be a measure of uncorrelated spike trains decoupling the memory from the discrimination condition. In more detail, the LTP part of STDP can be interpreted as a measure of the probability that the pre- and postsynaptic neurons fire correlated spikes during a small time window (Dayan & Abbott, 2001), described here in the rate-model by the correlation-based LTP-term, whereas the amount of uncorrelated spike pairs triggering LTD could be described in the here-used rate-model by the difference between the pre- and postsynaptic firing rates. We expect that considering such a difference term would be sufficient to enable the formation of memory representations(by correlation-based LTP) in a discrimination state (by non-correlation-based LTD). This has to be verified in subsequent studies.

However, given a population model incorporating detailed dynamics of correlation-based synaptic plasticity, the here-presented framework can be extended to investigate the influence of more complex stimulus protocols on the formation of diverse functional organizations. In a more realistic scenario, different stimuli could be presented in a probabilistic manner determined by different sources (hidden causes). The detection of several independent hidden sources from a stream of stimuli is a complex problem humans or cell cultures are able to solve (Mesgarani & Chang, 2012; Isomura, Kotani, & Jimbo, 2015). Several theoretical studies mainly focusing on neuronal networks with a feed-forward structure indicate that the dynamics of synaptic plasticity enables the solving of such types of problems (Dayan & Abbott, 2001; Bell & Sejnowski, 1995; Hyvärinen & Oja, 2000; Isomura & Toyoizumi, 2016; Pehlevan, Mohan, & Chklovskii, 2017; Isomura & Toyoizumi, 2018). Thus, we suppose that plastic feed-forward and recurrent connections enable the neuronal system to detect hidden sources by the feed-forward dynamics and forms memory representations of these by the recurrent synapses. Given relations between these sources, the synapses connecting the memory representations could represent the strength of these relations. Of course, if the number of sources increases, due to the increase in combinations of different functional organizations (Figure 8), the here-derived theoretical framework has to be adjusted. For instance, by measuring the mutual information or transfer entropy (Brunel & Nadal, 1998; MacKay, 2003; Vicente, Wibral, Lindner, & Pipa, 2011) conditioned on the input between representations, one could extract the formed functional organization given different stimulus protocols.

Beyond the scope of reliably forming memory representations of environmental stimuli, it is still unclear how to maintain these representations for a long duration (Dudai, 2004, 2012). Similar to previous studies (Tetzlaff et al., 2013, 2015), also here the interplay between synaptic plasticity and scaling yields the slow decay of synaptic weight structures after withdrawing the stimuli (see Supporting Information Figure S1 for different rates of synaptic dynamics; Herpich & Tetzlaff, 2019). However, as long as the average synaptic weight remains larger than control, the corresponding neurons and synapses resemble a memory representation of the stimulus. Prolonging the lifetime of such a memory can be done by the diverse mechanisms of consolidation as synaptic (Frey & Morris, 1997; Clopath, Ziegler, Vasilaki, Büsing, & Gerstner, 2008; Redondo & Morris, 2011; Li, Kulvicius, & Tetzlaff, 2016) or sleep-induced consolidation (Tetzlaff et al., 2013; Diekelmann & Born, 2010; Nere, Hashmi, Cirelli, & Tononi, 2013). We expect that similar mechanisms can also consolidate the intermemory synaptic weights maintaining the whole functional organization. However, under which conditions memory representations as well as the interconnections are consolidated requires further experimental and theoretical studies.

Please note that there is a multitude of studies indicating the existence of additional factors influencing synaptic plasticity. For instance, neuromodulatory transmitters, such as acetylcholine, noradrenaline, serotonin, and dopamine, can serve as a third factor (Frémaux & Gerstner, 2016; Gu, 2002). However, with the mathematical framework developed in this study, it is now possible to investigate in more detail the effect of such factors on the formation, maintenance, and organization of memory representations in neuronal circuits. Furthermore, given the understanding of the organization between two memory representations, now one can extend this framework to investigate the self-organized formation of webs of memories and the emergence of complex behavior.

## MATERIALS AND METHODS

### Neuronal Network Model

We consider a recurrent neuronal network model consisting of a set 𝒩 of *n* rate coded neurons (𝒩 ≔ ℕ_*n*_ {1, …, *n*}, Figure 2A, dots). The neurons are interconnected via an all-to-all connectivity for the excitatory as well as for the inhibitory connections. Note that, if not stated otherwise, the inhibitory connections are constant while the excitatory synapses are plastic. Within the recurrent network, we define two distinct subsets of neurons *𝒫1* and *𝒫2* as *neural population 1* (black dots) and *neural population 2* (yellow dots). For simplicity, both neural populations have the same number of neurons (|*𝒫1*| = |*𝒫2*| = *n_𝒫_*). Furthermore, we assume no overlap between both neuronal populations (*𝒫1* ∩ *𝒫2* = ∅). Neurons that are not part of neuronal population *𝒫1* or *𝒫2* are summarized as *background neurons* (*ℬ* ≔ 𝒩 ∖(*𝒫1* ∪ *𝒫2*)), with size |*ℬ* | = *n* −2*n*_*𝒫*_). Thus, we can describe the neuronal network model as the interaction of three different neuronal populations 𝒫 ∈ {*𝒫1*, *𝒫2*, *ℬ* }≕𝔅. All neurons *i* of a neuronal population *p* receive a population-*p*-specific input stimulation defined by *F*_*p*_^ex^ via *n*_*𝒫*_^ex^ different neurons *k* connected via constant excitatory synapses ω^ex^. All these input neurons *k* are summarized to a population-*p*-specific input *ε*_*p*_ ∈ *ε*. Furthermore, each single neuron *k* ∈ *ε*_*p*_ of the external input layer provides an external input stimulus of average strength *I_k_*^ex^ ≔ *F*_*k*_^ex^ ω^ex^ onto the interconnected neurons of the neuronal network 𝒩, where *F*_*k*_^ex^ is defined by the population-*p*-specific stimulation parameter *F*_*p*_^ex^ (see Equation 37). Note, we set *n*_*𝒫*_^ex^ equal to *n*_𝒫_ to consider the same order of magnitude for input populations as for the populations themselves.

#### Neuron model.

We consider point neurons with each neuron *i* ∈ *p* ⊂ 𝒩 of the network summing up its incoming inputs from the interconnected excitatory (Figure 2A, blue connections), inhibitory, and input neurons (red connections) to its overall neuron-specific input current (*ϕ*_*i*_). The inputs are transmitted via the synapses, thus, the neuron specific input current integrates the separate inputs of the neurons (*F*_*j*_) proportional to the respective synaptic weights (*ω*_*i*,*j*_):

$$\phi_{i}=\underset{\text{network exc.}}{\underset{︸}{\underset{j\in N_{i}^{+}}{\sum }\omega_{i,j}F_{j}}}-\underset{\text{network inh.}}{\underset{︸}{\underset{j\in N_{i}^{-}}{\sum }\omega_{i,j}^{-}F_{j}}}+\underset{\text{external exc.}}{\underset{︸}{\underset{k\in \varepsilon_{p}}{\sum }I_{k}^{\text{ex}}}},\;[\phi ]=1\text{pA}.$$ (24)

Here, 𝒩_*i*_^+^, 𝒩_*i*_^−^ ∈ 𝒩 are the sets of indices for the excitatory, inhibitory, and externally interconnected presynaptic neurons *j* to the postsynaptic neuron *i*, whereby, *ω*_*i*,*j*_ and *ω*_*i*,*j*_^−^([*ω*] = 1pC) are the respective synaptic weights, and *F*_*j*_([*F*] = 1s^−1^) the respective presynaptic neuron’s activity. In contrast to the plastic excitatory synaptic weights, all inhibitory synapses (θ : =*ω*_*i*,*j*_^−^ = constant) are constant. Although any neuron can have only either excitatory or inhibitory outgoing connections, similar to previous studies (Tetzlaff et al., 2013, 2015), we merge both distinct neuronal sets for the excitatory and inhibitory neurons to one neuronal population generalized by 𝒩 = 𝒩_*i*_^+^ = 𝒩_*i*_^−^. This approach is a simplification of the used model by assuming that an excitatory and an inhibitory neuron have a similar connectivity. This leads to the following input current (*ϕ*_*i*_) onto one neuron *i* belonging to population *p*:

$$\phi_{i}=\underset{j\in N}{\sum }(\omega_{i,j}-\theta )F_{j}+\underset{k\in \varepsilon_{p}}{\sum }I_{k}^{\text{ex}},\;\theta ,\omega^{\text{ex}}\in R_{+}.$$ (25)

We can further specify this input current in respect to the presynaptic neuron’s affiliation to a neuronal population 𝒫 ∈ {*𝒫1*, *𝒫2*, *ℬ* }:

$$\phi_{i}=\underset{p\in P}{\sum }\underset{j\in p}{\sum }(\omega_{i,j}-\theta )F_{j}+\underset{k\in \varepsilon_{p}}{\sum }I_{k}^{\text{ex}}.$$ (26)

This neuron specific input drives its respective membrane potential (u_*i*_) described by:

$$\tau {\overset{\cdot }{u}}_{i}=-u_{i}+\text{R}\phi_{i},\;\tau ,\text{R}\in R_{+},[u]=1\text{mV}.$$ (27)

Here, τ([τ] = 1s) is the time constant for the membrane potential and set to τ = 1s and R = 0.1nΩ,([R] = 1nΩ) is the membrane resistance. The membrane potential u_*i*_ is nonlinearly transformed to a neural firing rate (*F*_*i*_):

$$\begin{array}{l} F_{i}=\frac{{\text{F}}^{\text{max}}}{1+\exp[\beta (\epsilon -u_{i})]},\;{\text{F}}^{\text{max}},\beta ,\epsilon \in R_{+},\;[F]=1\text{Hz}, \end{array}$$ (28)

with F^max^ = 100Hz being the maximal firing rate, β([β] = 1mV^−1^) being the steepness, and ϵ([ϵ] = 1mV) being the inflexion point of the sigmoid. Thus, the neuronal activity for each neuron *i* takes values between 0 and F^max^(*F*_*i*_ ∈ [0,F^max^]). To simplify the description of the neuronal dynamics, we combine Equation 27 and Equation 28 to:

$$\begin{array}{ll} \tau {\overset{\cdot }{F}}_{i}= & \frac{({\text{F}}^{\text{max}}-F_{i})F_{i}}{{\text{F}}^{\text{max}}}\left[\log\left(\frac{{\text{F}}^{\text{max}}}{F_{i}}-1\right)+\beta (\text{R}\phi_{i}-\epsilon )\right]. \end{array}$$ (29)

#### Synaptic plasticity and synaptic scaling.

All excitatory synapses within the recurrent network are plastic and change proportional to the activity-dependent Hebbian learning rule (*H*; Hebb, 1949; Bliss & Lømo, 1973)

$$H:\;{\left(0,{\text{F}}^{\text{max}}\right)}^{2}\to R_{+},\;(F_{i},F_{j})\mapsto \mu F_{i}F_{j},\;\mu \in R_{+},$$ (30)

with time constant μ. This correlation learning rule leads to unbounded synaptic weight dynamics. Thus, we include synaptic scaling (*S*; Turrigiano et al., 1998; Turrigiano & Nelson, 2004) as a homeostatic mechanism

$$S:\;(0,{\text{F}}^{\text{max}})\times R_{+}\to R,\;(F_{i},\omega_{i,j})\mapsto \gamma ({\text{F}}^{\text{T}}-F_{i})\omega_{i,j}^{2},\;\gamma ,{\text{F}}^{\text{T}}\in R_{+},$$ (31)

with the time constant γ and target firing rate F^T^. This scaling mechanism decreases (increases) the synaptic weight if the postsynaptic activity is above (below) the target firing rate. Note that Hebbian dynamics are in general faster than scaling dynamics, thus, *γ* ≪ *μ*. Therefore, we set $\mu =\frac{1}{60\text{s}}$ on the timescale of minutes while $\gamma =\frac{1}{90\cdot 60\text{s}}$ on timescale of hours. Combining the correlation-based Hebbian learning term additive with the postsynaptic activity-dependent synaptic scaling term (*ω*·_*i*,*j*_ ∝ *H* + *S*), we get the following learning rule for the synaptic weights (Tetzlaff et al., 2011, 2012):

$$\begin{array}{l} {\overset{\cdot }{\omega }}_{i,j}=\mu F_{i}F_{j}+\gamma ({\text{F}}^{\text{T}}-F_{i})\omega_{i,j}^{2}. \end{array}$$ (32)

#### Constraining parameters of the activity function.

In the following, we give an interpretation for the parameters like the inflexion point and steepness of the sigmoidal shaped activity function (Equation 28). To specify the inflexion point of the neuronal activities, we define the maximal evoked membrane potential (*u*^max^) of a neuron *i* by only one incoming synapse (*ω*_*ij*_) to the postsynaptic neuron *j*. Therefore, we set the pre- and postsynaptic neuronal activities to the maximal activity level of (*F*_*j*_ = *F*_*i*_ =F^max^) and, by this, calculate the fixed synaptic weight, using Equation 32, and define it as the maximal synaptic weight ($\omega^{\text{max}}:=\sqrt{\mu {\left({\text{F}}^{\text{max}}\right)}^{2}/(\gamma ({\text{F}}^{\text{max}}-{\text{F}}^{\text{T}})}$). Equation 27 specifies the maximal network internal (∑*I*_*k*_^ex^ = 0) evoked membrane potential of $u^{\text{max}}:=\text{R}{\text{F}}^{\text{max}}(\omega^{\text{max}}-\tilde{\theta })$. Using this quality of *u*^max^, we interpret the inflexion ϵ of a neuron *i* as the number (n_ϵ_) of such maximally wired presynaptic neurons. This leads to ϵ =n_ϵ_*u*^max^. For the determination of the precise value for n_ϵ_ = 12 see the Results section. To specify the steepness of the neuronal activity function, we have to consider their maximal and minimal possible evocable membrane potential and choose a steepness parameter β because of two different constraints: (1) the activity for the minimal membrane potential has to take on higher values as the target firing rate F^T^ to prevent unstable weight dynamics, and (2) for a maximal evoked membrane potential the neurons have to take on the maximal firing rate of F^max^. One specific parameter for the steepness of the activity function that fulfill these two conditions is β = 0.00035mV^−1^ for all neurons.

### Normalized Neuronal Network Model

In the following, to reduce complexity, we normalize the neuronal activities of all neurons *i* ∈ 𝒩 according to the maximal neural firing rate (${\tilde{F}}_{i}:=F_{i}/{\text{F}}^{\text{max}}$) and all synaptic weights to the maximal excitatory synaptic weight (${\tilde{\omega }}_{i,j}:=\omega_{i,j}/\omega^{\text{max}};$$\tilde{\theta }:=\theta /\omega^{\text{max}}$). Thus, the external input stimulation is also normalized to ${Ĩ}_{k}^{\text{ex}}=I_{k}^{\text{ex}}/({\text{F}}^{\text{max}}\omega^{\text{max}})$. By this, we map the (normalized) neuronal activity ${\tilde{F}}_{i}$ and the (normalized) excitatory synaptic weight ${\tilde{\omega }}_{i,j}$ to [0,1] ∈ ℝ:

$$\tau {\overset{\cdot }{\tilde{F}}}_{i}=(1-{\tilde{F}}_{i}){\tilde{F}}_{i}\left[\log\left(\frac{1}{{\tilde{F}}_{i}}-1\right)+\beta (\text{R}\phi_{i}-\epsilon )\right]\;,$$ (33)

for the neuronal activity with

$$\phi_{i}={\text{F}}^{\text{max}}\omega^{\text{max}}\left(\underset{p\in P}{\sum }\underset{j\in p}{\sum }({\tilde{\omega }}_{i,j}-\tilde{\theta }){\tilde{F}}_{j}+\underset{k\in \varepsilon_{p}}{\sum }{Ĩ}_{k}^{\text{ex}}\right)\;,$$ (34)

and

$$\tau_{\omega }{\overset{\cdot }{\tilde{\omega }}}_{i,j}={\tilde{F}}_{i}{\tilde{F}}_{j}+\frac{{\tilde{\text{F}}}^{\text{T}}-{\tilde{F}}_{i}}{1-{\tilde{\text{F}}}^{\text{T}}}{\tilde{\omega }}_{i,j}^{2},$$ (35)

for the synaptic weight with

$$\tau_{\omega }={\tilde{\mu }}^{-1}={\left({\text{F}}^{\text{max}}\sqrt{{\text{F}}^{\text{max}}\mu \gamma (1-{\tilde{\text{F}}}^{\text{T}})}\right)}^{-1}\;.$$ (36)

### Numerical Simulation and Stimulation Protocol

Each neuronal simulation starts with a tuning phase, where each of the *n* = 100 neurons of the network receive input noise from *n*_*𝒫*_^ex^ = 10 different input neurons of the input layer ε. For the simulations in Figure 2 and Figure 3 the noise for the input neurons is determined by 𝒩 (0.25, 0.025) ⋅ F^max^. In all remaining simulations, we applied input noise of 𝒩 (0.05, 0.025) ⋅ F^max^. Subsequent to this tuning phase (*t* = 10), all neurons *k* of the external input layer *ε*_*p*_ that are connected to neurons *i* ∈ *p* fire according to an Ornstein-Uhlenbeck process:

$${\overset{\cdot }{\tilde{F}}}_{k}^{\text{ex}}=\underset{\text{drift}}{\underset{︸}{\delta ({\tilde{\text{F}}}_{\text{p}}^{\text{ex}}-{\tilde{F}}_{k}^{\text{ex}})}}+\underset{\text{diffusion}}{\underset{︸}{\frac{\sigma }{\text{d}t}\zeta_{k}}},\;\zeta_{k}∼N(0,1)$$ (37)

with a drift term with constant δ = 0.025 and an arbitrary population specific equilibrium level for the firing rate of ${\tilde{\text{F}}}_{\text{p}}^{\text{ex}}$, a normal distributed diffusion term with constant σ = 0.0125 and an initial firing rate of ${\tilde{F}}_{k}^{\text{ex}}(t=0)={\tilde{\text{F}}}_{\text{p}}^{\text{ex}}$. For values used for the inputs ${\tilde{\text{F}}}_{\text{p}}^{\text{ex}}$, please see captions of corresponding figures. Please note, as the comparison to the population model at equilibrium that is based on the average activities and average synaptic weights indicates (Figure 3), we expect that the specific type of noise does not significantly influence the results. We apply the input stimulation over time, by this we simulate the whole activity and synaptic weight dynamics until the system reaches an equilibrium state. Numerically, we solve the differential equations of the normalized model (Equations 33–35; with ${Ĩ}_{k}^{\text{ex}}={\tilde{\omega }}^{\text{ex}}\;{\tilde{\text{F}}}_{k}^{\text{ex}}$) for the synaptic weight dynamics and activity dynamics with the *euler method* ($\Delta t=\frac{1}{60}10^{-3}[\mu^{-1}]=1\text{ms}$) and parameters provided in Table 2; $\omega^{\text{max}}=\sqrt{\mu {\left({\text{F}}^{\text{max}}\right)}^{2}/(\gamma ({\text{F}}^{\text{max}}-{\text{F}}^{\text{T}})}$ and ϵ =n_ϵ_*u*^max^ with $u^{\text{max}}=R{\text{F}}^{\text{max}}(\omega^{\text{max}}-\tilde{\theta })$ and n_ϵ_ = 20 for Figures 2,3 and n_ϵ_ = 12 for Figure 7. All initial synaptic weights are distributed around the balanced state ${\tilde{\omega }}_{i,j}(t=0)∼N(\tilde{\theta },0.025)$ and activities around ${\tilde{F}}_{i}(t=0)∼N(0.07,0.005)$. In our model we also consider autapses *ω*_*ii*_.

**Table 2.** Used Parameters.

Network model		Neuron model		Syn. plast. & syn. scal.	
parameter	value	parameter	value	parameter	value

*n*	100 neurons	τ	1s^−1^	μ	$\frac{1}{60}{\text{s}}^{-1}$
					
$n_{P}$	10 neurons	R	0.1nΩ	γ	$\frac{1}{5400}{\text{s}}^{-1}$
					
*n*_*ℬ*_	$n-2n_{P}$	F^max^	100[Hz]	${\tilde{\text{F}}}^{\text{T}}$	0.05[F^max^]
					
$n_{P}^{\text{ex}}$	10 neurons	β	$0.00035\frac{1}{\text{mV}}$		
					
${\tilde{\omega }}^{\text{ex}}$	1				
					
$\tilde{\theta }$	0.5				

### Analysis of the System’s Equilibrium State

As the different functional organizations of two interconnected neuronal populations are defined at the system’s equilibrium state, we can reduce our problem to an analytical calculation of the average neuronal activities and synaptic weights at equilibrium state. Because of the homogeneous external input stimulation of all neurons *i* belonging to one neuronal population *p* and the underlying full connectivity of the network, we make the assumption that the fixed firing rate of each neuron *i* of a population *p* (${\tilde{F}}_{i}^{*}$) approaches the mean firing rate of the particular population (𝔉_*p*_) at fixed point state

$$F_{p}:=n_{p}^{-1}\underset{i\in p}{\sum }{\tilde{F}}_{i}^{*}\approx {\tilde{F}}_{i}^{*}.$$ (38)

Using these average activities (𝔉_*p*_) of both neuronal populations *p* at stable state, we can calculate the respective average excitatory synaptic weights at the system’s stable state

$$w_{p^{\prime }p}=\sqrt{\frac{F_{p}F_{p^{\prime }}\left(1-{\tilde{\text{F}}}^{\text{T}}\right)}{F_{p^{\prime }}-{\tilde{\text{F}}}^{\text{T}}}}.$$ (39)

This approach, reduces the problem to analytically calculate the average activities (𝔉_*p*_) of the neuronal populations 𝒫 ∈ {*𝒫1*, *𝒫2* } dependent on the external input stimulation. Therefore, we calculate the mean population specific external input current onto one neuron *i* of population p as ${Ĩ}_{p}^{\text{ex}}:=\sum_{k\in \varepsilon_{i},i\in p}{Ĩ}_{k}^{\text{ex}}$.

We easily see that the input current onto each neuron *i* of a neuronal population *p* is independent of the neuronal dynamics and is only defined by the average qualities of each neuronal population at the system’s stable state. Thus, the fixed mean input onto a neuronal population *p* (𝔔_*p*_) is given by:

$$\begin{array}{cc} \;Q_{p} & \approx {\text{F}}^{\text{max}}\omega^{\text{max}}\left(n_{P}{Ĩ}_{p}^{\text{ex}}+\underset{p^{\prime }\in P}{\sum }\underset{j\in p}{\sum }\left({𝔴}_{pp^{\prime }}-\tilde{\theta }\right)F_{p^{\prime }}\right)\\ & ={\text{F}}^{\text{max}}\omega^{\text{max}}\left(n_{P}{Ĩ}_{p}^{\text{ex}}+\underset{p^{\prime }\in P}{\sum }n_{P}\left({𝔴}_{pp^{\prime }}-\tilde{\theta }\right)F_{p^{\prime }}\right)\\ \overset{\text{Eq}\;39}{=} & {\text{F}}^{\text{max}}\omega^{\text{max}}n_{P}\left({Ĩ}_{p}^{\text{ex}}+\underset{p^{\prime }\in P}{\sum }\left(\sqrt{\frac{F_{p}F_{p^{\prime }}\left(1-{\tilde{\text{F}}}^{\text{T}}\right)}{F_{p}-{\tilde{\text{F}}}^{\text{T}}}}-\tilde{\theta }\right)F_{p^{\prime }}\right). \end{array}$$ (40)

We further reduce the complexity of the model to enable a fixed point analysis, while adding the fixed input from the background neurons onto one neuronal population *p* at stable state

$$\begin{array}{l} {Ĩ}_{pB}:=n_{P}(n-2n_{P})\left({𝔴}_{pB}-\tilde{\theta }\right)F_{B} \end{array}$$ (41)

to the external input stimulation (${Ĩ}_{p}^{\text{ex}}$), proportionally to the size of the external input layer ($\Delta {Ĩ}_{p}^{\text{ex}}:={Ĩ}_{pB}/n_{P}$). This leads to the total input current onto one neuronal population 𝒫 ∈ {*𝒫1*, *𝒫2* }), as:

$$\begin{array}{l} Q_{p}={\text{F}}^{\text{max}}\omega^{\text{max}}n_{P}\left(I_{p}+\underset{p^{\prime }\in \{P1,P2\}}{\sum }\left(\sqrt{\frac{F_{p}F_{p^{\prime }}\left(1-{\tilde{\text{F}}}^{\text{T}}\right)}{F_{p}-{\tilde{\text{F}}}^{\text{T}}}}-\tilde{\theta }\right)F_{p^{\prime }}\right), \end{array}$$ (42)

with $I_{p}:={Ĩ}_{p}^{\text{ex}}+\Delta {Ĩ}_{p}^{\text{ex}}$. This expression of the input current onto each neuronal population *p* at stable state allows us to numerically calculate the respective average activity of each population at the system’s stable state:

$$F_{p}^{\text{FP}}:=(1-F_{p})F_{p}\left(\log\left(\frac{1}{F_{p}}-1\right)+\beta (\text{R}Q_{p}-\epsilon )\right)\overset{!}{=}0$$ (43)

in a two dimensional parameter-phase space of ($I_{P1}-I_{P2}\;-$ space).

### Inhibitory Synaptic Plasticity

In the last section of this study, we introduce inhibitory synaptic plasticity. This specific plasticity rule depends on a threshold (θ_F_) for the sum of pre- and postsynaptic activity levels ($\sum F:={\tilde{F}}_{i}+{\tilde{F}}_{j}$) and on a tolerance range (*δF*) for the difference in the pre- and postsynaptic firing rates ($\Delta F:=|{\tilde{F}}_{i}-{\tilde{F}}_{j}|$) leading the inhibitory synaptic weight (${\tilde{\omega }}_{j,i}^{-}$) converge either to an up- (θ_u_) or down-state (θ_*d*_). The synaptic weight converge to:

• an up-state (θ_u_), if the sum of neuronal activities is smaller than its threshold (∑*F* < θ_F_) **and/or** the difference in the pre- and postsynaptic activities increases its tolerance range (Δ *F* > *δ F*),
• a down-state (θ_*d*_), if the sum of neuronal activities is greater than its threshold (∑ *F*> θ_F_) **and** the difference in the pre- and postsynaptic activities is smaller than its tolerance range (Δ*F* < *δF*).

These conditions lead to the following learning rule on the inhibitory synaptic plasticity:

$$\begin{array}{c} {\overset{\cdot }{\tilde{\omega }}}_{j,i}^{-}={\tilde{F}}_{i}{\tilde{F}}_{j}\left(\rho_{u}({\tilde{\theta }}_{u}-{\tilde{\omega }}_{ji}^{-}){\text{H}}^{\prime }[\text{H}(\Delta F-\delta \text{F})+\text{H}(\theta_{\text{F}}-\sum F)]\right.\\ \left.{+\rho }_{d}({\tilde{\theta }}_{d}-{\tilde{\omega }}_{ji}^{-})\text{H}(\delta \text{F}-\Delta \text{F})\text{H}(\sum F-\theta_{\text{F}})\right), \end{array}$$ (44)

with ρ_u_, ρ_*d*_ being the learning rates towards the up- and down-state and H*′* an adapted Heaviside function with H*′*(0) = 0 to express the and/or condition for the up-state. In our simulations we set θ_u_ = 0.8, θ_*d*_ = 0.5, θ_F_ = 2𝔉^min^, *δF* = 0.05, and ρ_*d*_ =ρ_*u*_ = 1.

## ACKNOWLEDGMENTS

The authors thank the International Max Planck Research School for Physics of Biological and Complex Systems, Niedersächsisches Voraband University of Göttingen, for a Stipend to Juliane Herpich.

## AUTHOR CONTRIBUTIONS

Juliane Herpich: Conceptualization; Formal analysis; Investigation; Methodology; Visualization; Writing - Original Draft. Christian Tetzlaff: Conceptualization; Funding acquisition; Methodology; Project administration; Supervision; Validation; Writing - Review & Editing.

## FUNDING INFORMATION

Christian Tetzlaff, H2020 Future and Emerging Technologies (http://dx.doi.org/10.13039/100010664), Award ID: 732266. Christian Tetzlaff, Deutsche Forschungsgemeinschaft (http://dx.doi.org/10.13039/501100001659), Award ID: SFB-1286, Project C1.

## TECHNICAL TERMS

Synaptic plasticity: General term for all kind of different biological mechanisms adapting the weights of synapses. Often they depend on neuronal activities.
Cell assembly: A group of neurons being strongly interconnected and essentially active together.
Correlation-based synaptic plasticity: Synaptic plasticity mechanisms adapting synaptic weights depending on the correlation of the pre- and postsynaptic neuronal activities.
Homeostatic plasticity: Synaptic plasticity mechanism adapting the synaptic weights such that neuronal systems maintain a desired average activity level.
Synaptic weights: The average transmission efficacy of a synapse quantified as a single number, which can be adapted by synaptic plasticity.
