# Supplementary material for: Principles underlying the input-dependent formation and organization of memories
Source: Netw Neurosci. 2019 May 1;3(2):606–34. doi: 10.1162/netn_a_00086 (PMC6542621; doi:10.1162/netn_a_00086)
Supplement: Supplementary file 1 [file netn-03-606-s001.pdf]

Herpich, J. & Tetzlaff, C. (2019). Supporting Information for "Principles underlying the input-dependent formation and organization of memories." *Network Neuroscience*, *3*(2), 606-634. https://doi.org/10.1162/netn_a_00086

SUPPLEMENT

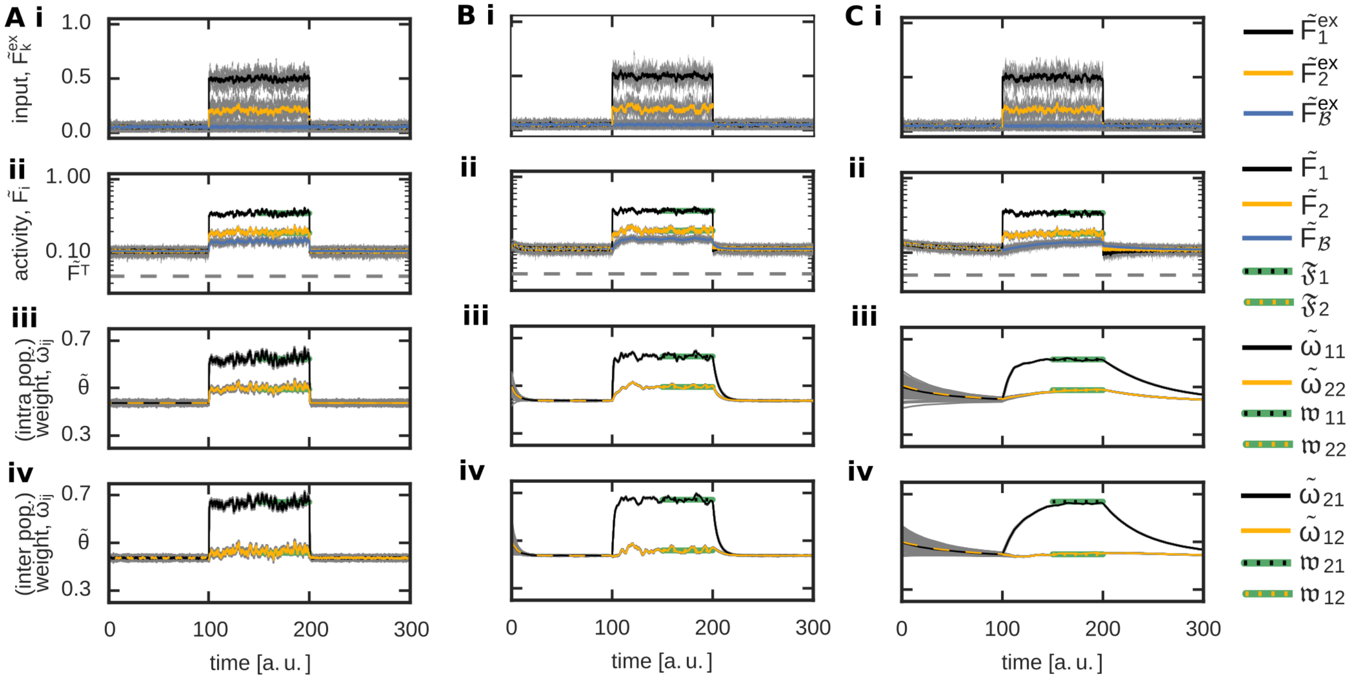

Suppl. Fig. 1. Numerical simulation of two interconnected neuronal populations in the network model with different synaptic learning rates considering the tuning, stimulation and after-stimulation phase. The input stimulation of both neuronal populations is $\tilde{F}^{ex}_{p_1} = 0.5$ and $\tilde{F}^{ex}_{p_2} = 0.2$ for all three simulations. **A** $\mu = \frac{1}{60}$. **B** $\mu = \frac{1}{1{,}000}$. **C** $\mu = \frac{1}{10{,}000}$. For all simulations holds $\gamma = \frac{\mu}{90}$.
